# Distribution of nitrogen fixation and nitrogenase-like sequences amongst microbial genomes

**DOI:** 10.1186/1471-2164-13-162

**Published:** 2012-05-03

**Authors:** Patricia C Dos Santos, Zhong Fang, Steven W Mason, João C Setubal, Ray Dixon

**Affiliations:** 1 Department of Chemistry, Wake Forest University, Winston-Salem, NC, USA; 2 Virginia Bioinformatics Institute, Virginia Tech, Blacksburg, VA, USA; 3 Departmento de Bioquímica, Instituto de Química, Universidade de São Paulo, São Paulo, SP, Brazil; 4 Department of Molecular Microbiology, John Innes Centre, Norwich Research Park, Colney, Norwich, NR4 7UH, UK

## Abstract

**Background:**

The metabolic capacity for nitrogen fixation is known to be present in several prokaryotic species scattered across taxonomic groups. Experimental detection of nitrogen fixation in microbes requires species-specific conditions, making it difficult to obtain a comprehensive census of this trait. The recent and rapid increase in the availability of microbial genome sequences affords novel opportunities to re-examine the occurrence and distribution of nitrogen fixation genes. The current practice for computational prediction of nitrogen fixation is to use the presence of the *nifH* and/or *nifD* genes.

**Results:**

Based on a careful comparison of the repertoire of nitrogen fixation genes in known diazotroph species we propose a new criterion for computational prediction of nitrogen fixation: the presence of a *minimum set* of six genes coding for structural and biosynthetic components, namely NifHDK and NifENB. Using this criterion, we conducted a comprehensive search in fully sequenced genomes and identified 149 diazotrophic species, including 82 known diazotrophs and 67 species not known to fix nitrogen. The taxonomic distribution of nitrogen fixation in Archaea was limited to the Euryarchaeota phylum; within the Bacteria domain we predict that nitrogen fixation occurs in 13 different phyla. Of these, seven phyla had not hitherto been known to contain species capable of nitrogen fixation. Our analyses also identified protein sequences that are similar to nitrogenase in organisms that do not meet the minimum-gene-set criteria. The existence of nitrogenase-like proteins lacking conserved co-factor ligands in both diazotrophs and non-diazotrophs suggests their potential for performing other, as yet unidentified, metabolic functions.

**Conclusions:**

Our predictions expand the known phylogenetic diversity of nitrogen fixation, and suggest that this trait may be much more common in nature than it is currently thought. The diverse phylogenetic distribution of nitrogenase-like proteins indicates potential new roles for anciently duplicated and divergent members of this group of enzymes.

## Background

Biological nitrogen fixation is the major route for the conversion of atmospheric nitrogen gas (N_2_) to ammonia [1]. However, this process is thought be limited to a small subset of prokaryotes named diazotrophs, which have been identified in diverse taxonomic groups [2]. This biochemical pathway is only manifested when species-specific metabolic and environmental conditions are met, thus making it difficult to develop a standard screen for detection of this biological reaction [3,4]. The complications in experimentally detecting nitrogen fixation may be a reason for the relatively low number and relatively sparse distribution of known diazotrophic species.

All known diazotrophs contain at least one of the three closely related sub-types of nitrogenase: Nif, Vnf, and Anf. Despite differences in their metal content, these nitrogenase sub-types are structurally, mechanistically, and phylogenetically related. Their catalytic components include two distinct proteins: dinitrogenase (comprising the D and K component proteins) and dinitrogenase reductase (the H protein) [1,2]. The only known exception to this rule is the superoxide-dependent nitrogenase from *Streptomyces thermoautotrophicus*, whose protein sequence is unknown [5].

The best studied sub-type is the molybdenum-dependent (Mo-dependent) nitrogenase, the structural components of which are encoded by *nifH, nifD*, and *nifK*[1]. The two other sub-types of nitrogenase, known as alternative nitrogenases, are enzyme homologs with the exception of an additional subunit (G) in the dinitrogenase component and the absence of the heteroatom Mo. The vanadium-dependent nitrogenases are encoded by *vnfH, vnfD, vnfG*, and *vnfK*. The members of the third sub-type, the iron-only nitrogenases, are devoid of Mo and V, and their components are products of *anfH, anfD, anfG*, and *anfK*. High levels of protein sequence identity among analogous subunits across the nitrogenase sub-types allow investigation of the biodiversity in nitrogen fixation using NifH (similar to VnfH and AnfH) and/or NifD (similar to VnfD and AnfD) as markers. Most phylogenetic studies of nitrogen fixing organisms have used only NifH and/or NifD sequences as queries to assess diversity [4,6-8].

The high level of complexity of nitrogenase metalloclusters results in a laborious pathway for the assembly and insertion of the active site metal-cofactor, FeMoco, into dinitrogenase. Apart from the catalytic components, additional gene products are required to produce a fully functional enzyme [9]. Although the number of proteins involved in the activation of nitrogenase seems to be species-specific and varies according to the physiology of the organism and environmental niche [10,11], so far over a dozen genes have been identified as being involved in this process. Despite variations in the precise inventory of proteins required for nitrogen fixation, it is well acknowledged that the separate expression of the catalytic components is not enough to sustain nitrogen fixation, thus indicating that the FeMoco biosynthetic enzymes play a crucial role in dinitrogenase activation [12].

In the last few years, substantial advances have been made in the functional assignment of individual gene products involved in the biosynthesis of FeMoco in *Azotobacter vinelandii*[9,12,13]. The current biosynthetic scheme involves a consortium of proteins that assembles the individual components, iron and sulfur, into Fe-S cluster modules for subsequent transformation into precursors of higher nuclearity, and addition of the heteroatom (Mo) and organic component (homocitrate). The synthesis of FeMoco is completed in a so-called scaffold protein, NifEN, and shuttled to the final target by cluster carrier proteins. Interestingly, the scaffold NifEN has amino acid sequence similarity to NifDK [14].

The recent growth of genomic databases now including nearly 2,000 completed microbial genomes motivated us to re-evaluate the diversity of species capable of nitrogen fixation. Identification of co-occurrence of nitrogen fixing genes in species known to fix nitrogen enabled us to identify novel potential diazotrophs based on their genetic makeup. Our findings expand the expected occurrence of nitrogen fixation and the biodiversity of diazotrophs. In addition we have identified a large number of phylogenetically diverse nitrogenase-proteins that may represent ancestral forms of the enzyme and may have evolved to perform other metabolic functions.

## Results

### Species containing NifD and NifH-like sequences

The rapid expansion of microbial genome sequencing in the last few years affords novel opportunities to re-examine the distribution of nitrogen fixation genes. In this work, we have searched the genome sequences of fully sequenced microbe genomes available in GenBank [15] for coding sequences similar to NifD and NifH. The initial search included 1002 Archaeal and Bacterial distinct species with fully sequenced genomes, 174 of which contained sequences similar to NifH as well as sequences similar to NifD. Literature searches on these species indicated that nitrogen fixation has not been experimentally demonstrated in more than half of these (92 out of 174), thus suggesting that the phylogenetic distribution of diazotrophs is wider than currently known. Based on the literature survey (Additional file 1: Table S1), we classified species with hits into two categories: (1) known diazotrophs - with experimental demonstration, and (2) potential diazotrophs - with no reports of experimental demonstration. Interestingly, during this literature search we found three recent reports providing experimental demonstration of diazotrophy motivated by an initial genomic identification of putative nitrogen fixation genes [16-18].

### Identification of a minimum gene set

**Figure 1:**
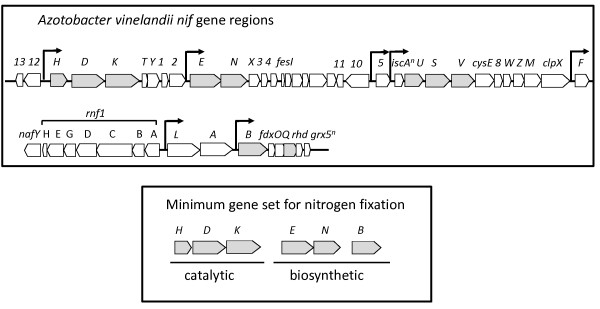
**Genes involved in nitrogen fixation.** Top- *A. vinelandii nif* gene regions. Gray-shaded trapezoids are essential genes in Mo-dependent nitrogen fixation that were used as queries for the *in silico* identification of nitrogen fixing species described in this study. Bottom –The proposed minimum set of genes required for nitrogen fixation. All species with sequenced genomes that are known diazotrophs and all the species proposed to be diazotrophs based on genetic content contain the minimum gene set.

The crucial involvement of the FeMoco biosynthesis enzymes prompted us to analyze the occurrence of nine additional *nif* genes in known diazotrophic species encoding NifK, NifE, NifN, NifB, VnfG, NifQ, NifV, NifS, and NifU. The involvement of eight of these proteins in FeMo-cofactor synthesis and nitrogenase maturation has been determined [3,9,12]. The co-occurrence of additional *nif* genes varied from species to species [19,20]. These differences in genetic requirements most probably reflect variations in meeting the physiological demands associated with nitrogen fixation and in species-specific metabolic and environmental life styles. Nevertheless, the identification of relevant hits (listed in the Additional file 2: Table S2) revealed that nearly *all* known diazotrophs contain a minimum of six conserved genes: *nifH, nifD, nifK, nifE, nifN,* and *nifB* (Figure 1). The co-occurrence of these six *nif* genes, known to be essential for nitrogen fixation in characterized systems, has led us to propose a requirement for a *minimum gene set* for nitrogen fixation that can be used as an *in silico* search tool for the identification of additional diazotrophs. We did find a few exceptions to this minimum gene set rule, and they are discussed below.

Our investigation showed that a clustered genomic arrangement of *nif* genes was a recurring feature in known diazotrophic genomes. In several species the minimum gene set was located in a single genomic region. In all cases, at least three out of the six genes contained in the minimum set were in contiguous gene regions. Most often, *nifHDK* were clustered, but in some other cases, *nifDK* was adjacent to *nifEN*. Nevertheless, the genomic synteny of *nif* genes across nitrogen-fixing species facilitated *in silico* assignments of putative sequences involved in nitrogen fixation.

### Identification of new diazotrophs

We identified potential diazotrophic species by computational searches using the minimum gene set (Additional file 2: Table S3). We identified 92 species containing coding sequences similar to NifD and NifH, 67 of which met the minimum gene set criteria (i.e. their genome contained at least *nifH, nifD, nifK, nifE, nifN*, and *nifB*). Based on gene content, we propose that these 67 species have the capacity for nitrogen fixation.

### Biodiversity of nitrogen fixing species

**Figure 2:**
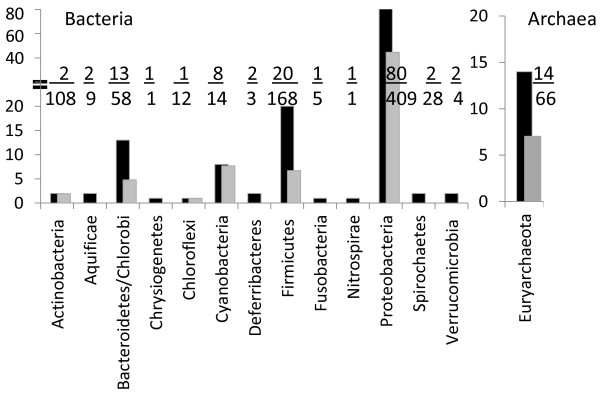
**Taxonomic diversity of nitrogen fixing species.** Species with fully sequenced genomes (999 Bacteria and 93 Archaea genomes) were analyzed for the minimum set of nitrogen fixation ortholog genes. Taxonomic distribution of diazotrophic species based on experimental evidence (gray bars) and *in silico* prediction of nitrogen fixation (black bars) is displayed by phylum. The ratio of the number of proposed species versus the number of total distinct species with sequenced genomes within each phylum is indicated.

The taxonomic distribution of diazotrophs identified through computational assignment suggests that nitrogen fixation has greater biodiversity. Prior to this work, known bacterial diazotrophs were found in six taxonomic phyla: Actinobacteria, Chlorobi, Chloroflexi, Cyanobacteria, Firmicutes and Proteobacteria (Figure 2 – gray bars). Our study resulted in the identification of potential diazotrophs within the already identified phyla and added seven new phyla (Figure 2 – black bars). Thus, despite the availability of few representatives in these other seven phyla (Figure 2), applying the minimum gene set criteria has expanded the biodiversity of this metabolic trait by approximately two-fold. No potential diazotrophs were identified in Acidobacteria (5), Deinococcus-Thermus (13), Dictyoglomi (2), Elusimicrobia (1), Fibrobacteres (1), Gemmatimonadetes (1), Planctomycetes (5), Synergistetes (2), Tenericutes (29), Thermotogae (12), Thermodesulfobacteria (3), and Thermomicrobia (1) (in parenthesis, the number of species in each group with fully sequenced genomes). The lack of diazotrophs within these phyla could be attributed to the under-representation of sequenced genomes in these taxonomic groups. Unlike bacterial species, nitrogen fixation in Archaea is contained only within the phylum Euryarchaeota, where we identified seven species as potential diazotrophs.

### Sporadic occurrence of alternative nitrogenase

The presence of an additional subunit, AnfG or VnfG (Additional file 2: Table S2, Additional file 2: Table S3) and distinct sequence features of alternative nitrogenases allowed us to distinguish the Mo-dependent enzymes from the alternative systems [3,21]. The genomes of most diazotrophs encode only one copy of the Mo-dependent sub-type of nitrogenase (134 out of 149 species). Exceptions were species containing additional sub-types (Vnf and/or Anf), such as the well-studied *A. vinelandii* and *Rhodopseudomonas palustris,* as well as *Dickeya dadantii, Chloroherpeton thalassium, Methanobacterium sp., Paludibacter propionicigenes, Rhodomicrobium vannielii, and Syntrophobotulus glycolicus*. Unexpectedly, selected Alphaproteobacteria species, including *Rhizobium etli* and *Sinorizobium fredii*, encoded two putative copies of Mo-dependent nitrogenase, where one copy of *nifHDK* is clustered with *nifEN* and the other copy only has genes similar to the catalytic components *nifHDK*. As previously proposed [10], alternative nitrogenases were only found in species containing genes coding for the Mo-dependent enzyme. This finding suggests that the hierarchy of expression of Mo-dependent over alternative nitrogenase, observed in *A. vinelandii,* may be universal to all species containing alternative nitrogenases [10].

### Phylogenetically distinct NifDK enzymes are present in thermophilic strains lacking a defined FeMoco biosynthesis pathway

**Figure 3:**
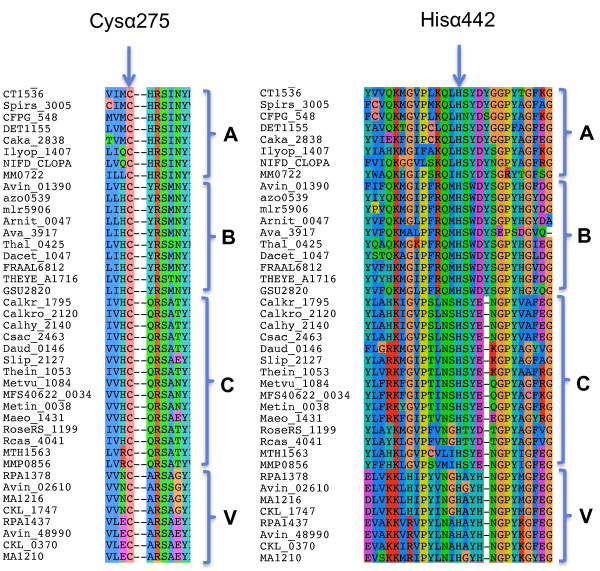
**Alignment of residues flanking conserved FeMoco ligands in NifD/VnfD/AnfD proteins.** Alignment of residues flanking the conserved co-factor ligands, Cys275 and His442, in the alpha subunit of Mo-dependent and alternative nitrogenases. (The sequence numbering refers to *A. vinelandii* NifD, Avin_01390.) Protein groups labeled A and B correspond to subfamilies 2 and 1 respectively, previously identified by Kechris et al. [23]. Group C represent the additional sub family described in the text. Group V corresponds to AnfD and VnfD sequences.

Our analysis of *nif* gene content revealed 28 strains that did not meet the minimal gene set criteria because they lacked either NifN or both NifE and NifN. Nevertheless, some of the hyperthermophilic representatives of this class, for example, the deep-sea vent archaeon *Methanocaldococcus* sp. FS406-22, have been demonstrated to fix nitrogen [22]. To further analyse the properties of the putative nitrogenases encoded by this class, we examined the environment of the FeMoco ligands in 15 NifD proteins, which we refer to collectively as group C. NifDK homologs belonging to this group possess the conserved Cys residues required for liganding a P cluster, and the NifD component contains the FeMoco ligands αCys275 and αHis442. The NifD subunits also contain the equivalents of αGln191 and αHis195 that are important for nitrogen reduction, and in addition, the homocitrate “anchor ligand” αLys426. Previous analysis identified two distinct subfamilies of NifD proteins (indicated as A and B in Figure 3) characterised by distinctive sequences surrounding their FeMoco ligands at αCys275 and αHis442 [23]. Group C represent a third subfamily, containing Gln at position 276, Asp at position 440 and lacking a residue corresponding to the aromatic amino acid found at position 444 in the A and B subfa-milies (Figure 3). Sequences in the C group are distinct from the alternative nitrogenase VnfD and AnfD subunits, which contain a conserved Ala at position 276, and a His residue replacing an acidic amino acid at position 445 (indicated as Group V in Figure 3).

The division of NifDK into three primary lineages, distinct from AnfD/VnfD/AnfK/VnfK is supported by phylogenetic analysis ([24] and Additional file 3: Figure S1). The existence of two lineages within conventional NifDK proteins has been shown to correlate with the domain structure of NifB in Bacterial and Archaeal proteins [25]. The third lineage (denoted as C in Additional file 3: Figure S1), entirely comprised of representatives of the Archaea and Firmicutes, appears to correlate with the absence of NifN and the sequence environment of the co-factor ligands in NifD. Notably the NifDK homologs in this lineage are all derived from thermophiles with the exception of *Methanococcus aeolicus Nankai-3,* which possesses both NifE and NifN. Two other NifDK sequences listed in the C group (Additional file 2: Table S3) are derived from the diazotrophic methanogens*, Methanobacterium thermoautotrophicum* Delta H, and *Methanococcus maripaludis* S2, which also encode *nifE* and *nifN*. The latter two NifDK proteins belong to a distinct group (labelled M in Additional file 3: Figure S1) that is considered to have emerged before all other nitrogenase proteins [24]. Thermophilic *Roseiflexus* species that lack both NifE and NifN also belong to a separate phylogenetic group (labelled R in Additional file 3: Figure S1). In conclusion, there is evidence for nitrogen fixation in species lacking *nifN*, but this appears to be associated with a thermophilic lifestyle and the presence of a phylogenetically distinct form of nitrogenase. Although this represents a clear exception to the minimal gene set, it appears to be a special case connected with the need to fix nitrogen in extreme environments.

### Nitrogenase-like sequences

**Figure 4:**
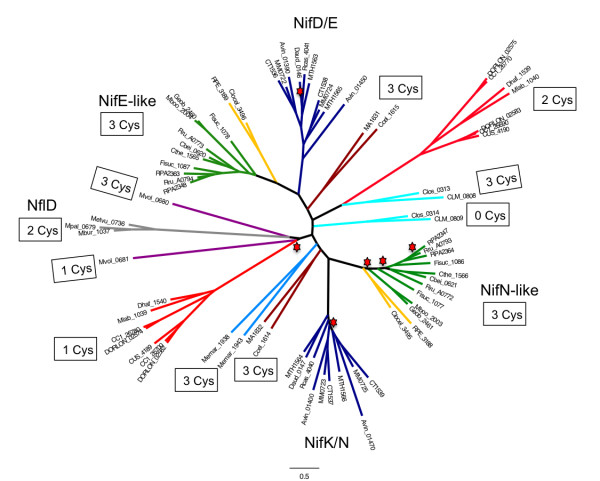
**Maximum-likelihood phylogenetic tree of conventional nitrogenases and nitrogenase-like sequences.** The tree is represented by a core set of 73 sequences, selected from a larger tree of 472 sequences. Shimodaira-Hasegawa local support values were >0.6 except for those nodes marked with a red star. The clade coloring reflects sequences that are co-located in genomes and likely to correspond to the alpha and beta subunits of nitrogenase, with the exception of those shown in light gray, which are single subunit enzymes (NflD). Dark blue clades are conventional nitrogenases, labeled as NifD/E and NifK/N respectively. Clades colored in light-green are NifD/E and NifK/N-like sequences in which the FeMoco ligand Cys 275 in the alpha component, is either present (dark green nodes) or absent (yellow nodes). In all other cases known FeMoco ligands are absent. The number of conserved Cys residues in each subunit that correspond to P cluster ligands in conventional nitrogenases are indicated for each clade.

During our search for nitrogenases we encountered a large number of proteins that appeared to be distantly related to the alpha and beta subunits of nitrogenase, but nevertheless belong to the Pfam nitrogenase component 1 type oxidoreductase family (PF00148). This Pfam family currently contains 2561 sequences, although a large proportion of these show similarity to the B and N subunits of the light-independent chlorophyllide reductase (DPOR), which is structurally related to Mo-Fe protein of nitrogenase. This enzyme does not contain a heterometal cluster analogous to FeMoco within its active site, and the co-ordination of the [4Fe4S] “NB” cluster within DPOR is different to that of the [8Fe7S] P cluster in nitrogenase [26]. After removal of DPOR-related sequences from our analysis by running a BLAST search against ChlB, BchB, ChlN and BchN, we observed that NifDK paralogs are represented in both diazotrophic and non-diazotrophic strains. Phylogenetic analysis of the BLAST-filtered subset revealed distinct groupings that are clearly divergent from conventional nitrogenase (Figure 4). These outgroups are also distinct from the DPOR enzymes, which form a separate clade (not shown in Figure 4). The existence of an outgroup of nitrogenase homologs (termed Group IV) has been noted previously [27], but the current availability of genome sequences has enabled more extensive analysis. It is highly unlikely that any of these nitrogenase-like proteins are competent to reduce dinitrogen as they lack ligands required to co-ordinate Fe-Moco.

Representatives of these non-conventional enzymes cluster in distinct clades relative to the conventional NifDKEN, Vnf/AnfDK and the C-group DK proteins, which are coloured dark blue in Figure 4. The genes encoding these non-conventional proteins are adjacent in genomes and have the potential to encode the alpha and beta subunits of nitrogenase-like enzymes. The lineages coloured either green or yellow in Figure 4 comprise groups of NifE or NifN related proteins that each contain the three conserved Cys residues involved in liganding the P cluster. The NifE-related subunits of partners coloured in green possess the FeMoco–ligand Cys275, but lack the highly conserved co-factor ligand, His 442. Those coloured in yellow lack both FeMoco ligands. It is possible that these proteins ligand an [4Fe–4S] cluster in a similar location to the P cluster in nitrogenase that delivers electrons to the active site. By analogy to NifEN, these enzymes may be able to reduce substrates with a limited number of electrons such as acetylene and azide [28]. These orthologs are found in diverse organisms, including the Proteobacteria, Archaea, Firmicutes and Fibrobacteres. Some organisms have an unusually large number of nitrogenase-like proteins of this class. For example, *Syntrophobotulus glycolicus* DSM 8271 contains nine protein pairs related to the alpha and beta subunits of nitrogenase. In two cases, these are organised as four linked genes (Sgly_0993, Sgly_0994, Sgly_0995 Sgly_0996 and Sgly_2775, Sgly_2776, Sgly_2777 and Sgly_2778) potentially located in operons, suggesting that some of these gene pairs may provide scaffolding functions for co-factor assembly into the structural subunits, analogous to the *nifDKEN* gene clusters encoding conventional nitrogenase.

More diverse representatives of the nitrogenase-like sequences are found in the Archaea and Firmicutes. These proteins lack FeMoco ligands and contain a variable number of conserved cysteine residues that may ligand a [Fe-S] cluster. For example *Clostridium botulinum* strains and *Alkaliphilus oremlandii* encode NifEN-like sequences (coloured light blue in Figure 4) that are located downstream of genes encoding NifH and a potential ATPase component of the ABC transporter family. Their NifE-related components (CLM_0808 and Clos_0313) contain the three conserved P cluster ligands, but conserved Cys residues are not present in the NifN-like components (CLM_0809 and Clos_0314). In contrast, *Methanocorpusculum labreanum* Z and *Desulfitobacterium hafniense* DCB-2 encode proteins with two conserved Cys residues (corresponding to αC88/αC62 and αC154/αC124) in the NifD/E-related components (Mlab_1040 and Dhaf_1539) and only a single conserved Cys residue (corresponding to ßC95/ßC44) in the NifK/N related subunits (Mlab_1039 and Dhaf_1540). Representative species from the Human Microbiome project, including *Coprococcus catus* GD/7 and *Dorea longicatena* DSM 13814, also appear in these clades (coloured red in Figure 4) and possess nitrogenase-like sequences with a similar arrangement of conserved cysteines. These organisms encode two closely linked copies of NifHEN-like sequences in their genomes. It is possible that a residue other than cysteine serves to co-ordinate an [Fe-S] cluster in representatives of these clades, as observed in the case of DPOR, which utilises an aspartate residue as a cluster ligand [26].

A variation in the arrangement of the subunits in these nitrogenase-like sequences is observed in some representatives of the Archaea, Firmicutes and Deltaproteobacteria, whereby *nifH* and *nifE*–like genes are fused to form a single open reading frame that is followed by a *nifN*-like gene (data not shown). In contrast, several representatives of the Archaea possess only a single gene encoding a homolog of the alpha and beta chains of nitrogenase (e.g. Metvu_0736, MpaI_0679 and Mbur_1037) (coloured grey in Figure 4). These form part of the outgroup identified by Raymond et al. [27] and are designated as NflD. These single subunit enzymes contain conserved Cys residues (corresponding to αC88/αC62 and αC154/αC124 in NifD/E) and are frequently annotated as putative methanogenesis marker 13 metalloproteins, which are thought to function in methanogenesis.

## Discussion

Biological nitrogen fixation is thought to be one of the most ancient enzyme-catalyzed reactions [27]. The elaborate architecture of its catalyst, which supports a complex reaction mechanism for dinitrogen reduction, has long been the subject of interest, not only from the viewpoint of evolutionary perspective and system complexity, but also as a fundamental biological process that can be exploited to develop new strategies for agricultural soil fertilization. The unpredictable occurrence of this metabolic trait across taxonomic groups, combined with the challenge of experimental detection of nitrogen fixation, makes it difficult to obtain a comprehensive census of prokaryotes with the capacity for diazotrophy.

The universal presence of gene sequences coding for the nitrogenase catalytic components in diazotrophs (*nifH* and *nifD*) is commonly used as a search tool in many phylogenetic studies. However, when using a single-gene survey in the database of microbial sequenced genomes, we detected orphan false-positive hits in several non-diazotrophic genomes. For example, the *Methanobrevibacter ruminantium M1* and *Methanocaldococcus fervens AG86* genomes include only a sequence similar to NifH, while the *Methanosphaera stadtmanae DSM 3091* genome contains only a NifD-like sequence. In this case orphan *nifD*-like sequences may be evolutionary relics of divergent enzymes in which the NifD/E component does not contain conserved FeMoco ligands (see below). Thus genome analysis of environmental samples based purely on BLAST hits to NifH or NifD may lead to false indications of diazotrophy. To eliminate hits from orphan sequences our initial approach was to search *in silico* for the co-occurrence of NifH and NifD and then subsequently filter these hits for the occurrence of other nitrogen fixation protein sequences.

Many previous studies have focussed on NifH and NifD sequences as markers for the phylogenetic distribution of diazotrophs. However, BLAST searches at relatively low threshold identified nitrogenase-like sequences lacking FeMo-co ligands (Figure 4).

False positives can therefore be obtained if only NifH and NifD are used in the search criteria. Extending the gene set to NifHDK or even to NifHDKB can also give rise to false positives, because sequences similar to the α and ß subunits of nitrogenase can be associated with NifH-like and NifB-like genes (Additional file 4: Figure S2). The strict requirement of a separate set of proteins involved in the assembly and synthesis of the active site cofactor, FeMoco, provides strong indication that the presence of *nifH* and *nifD* coding sequences alone does not provide enough evidence for diazotrophy. Therefore, our rationale was first to determine the inventory of *nif* genes that were *always* present in known-diazotrophic species. Literature searches combined with BLAST analyses led to the proposal that nitrogen fixation requires at least 6 gene products (Figure 1). Using this criterion, we found 67 species that we hypothesize have the metabolic capacity for nitrogen fixation. Our computational assignments provide a good indication that these species are potential diazotrophs and give direction to experimentalists to validate these predictions.

Our *in silico* assignments predict that nearly 15% of prokaryotic species with sequenced genomes are either known or potential diazotrophs, a fraction much larger than commonly accepted. The biased distribution of sequenced genomes in relation to taxonomic groups probably undermines a robust evaluation of the taxonomic diversity of nitrogen fixation in nature. For example, the phylum Proteobacteria has 409 genomes from distinct species, while Thermomicrobia is represented by only one. Efforts towards detailed functional assignments of biochemical pathways were also compatible with our findings. The SEED database [29] lists the occurrence of 20 *nif* genes in 45 unique species, and in all cases the minimum gene set is present. Almost all of these species are included in this study, the only exception being *Magnetospirillum gryphiswaldense*, which was not in the NCBI database of completed sequenced genomes at the time this study was completed. It is probable that nitrogen fixation also occurs in many other diverse species in which phyla are underrepresented in current databases. Therefore, applying the minimum gene set to newly sequenced genomes as they become available can lead to the identification of many other diazotrophs and further expand the diversity of diazotrophs in terms of taxonomic distribution of this metabolic trait.

Our study revealed a set of species for which our criteria for *in silico* prediction of nitrogen fixation were not satisfied, as they lack NifEN but nevertheless retain the nitrogenase structural genes together with *nifB* and *nifV.* Paradoxically*,* recent phylogenetic analysis suggests that NifDK homologs present in strains lacking NifN, such as *Caldicellulosiruptor saccharolyticus, Candidatus Desulforudis audaxviator* and *Methanocaldococcus* sp. FS406-22, emerged after the ancestral Mo enzymes found in hydrogenotrophic methanogens such as *M. maripaludis*, which have a complete FeMoco assembly pathway represented by early branching lineages of NifE and NifN [24,25]. Nevertheless, the uncharacterised nitrogenases belonging to the C group appear to have evolved prior to the emergence of most NifDK homologs in both Archaea and Bacteria. Our studies indicate that although the catalytic components contain structural motifs competent to coordinate FeMoco, these proteins have a distinct environment surrounding their co-factor ligands, which may confer unique maturation or catalytic properties. The presence of diazotrophic species within this group suggests that these nitrogenases may have distinct characteristics that permit a more parsimonious mechanism for FeMoco assembly. Without exception, organisms in the C-group that lack either NifN or NifEN are thermophiles inhabiting diverse environmental niches. Biochemical studies that mimic the absence of NifEN demonstrate that a NifDK enzyme containing NifB-co rather than FeMoco, exhibits hydrogen evolution and retains some ability to reduce acetylene, but not dinitrogen. Addition of molybdenum and homocitrate to the NifB-co containing enzyme did not influence substrate reduction [30]. Potentially, however, thermal adaptation might permit the assembly of FeMoco on a modified scaffold or perhaps on the NifDK subunits themselves. Further characterisation of nitrogen fixation and the properties of nitrogenase in these thermophilic organisms will be required to establish if FeMoco can indeed by assembled via an alternative route.

Our studies have highlighted a number of nitrogenase-like homologs belonging to oxidoreductase/nitrogenase component 1 family, which may have different metabolic functions compared to the well-characterised canonical representatives, nitrogenase and protochlorophyllide reductase. Structural studies reveal that the fold of these two enzymes is remarkably similar, with equivalent positioning of the [Fe-S] clusters enabling a similar mechanism of ATP-driven electron transfer from the reductase protein, to the catalytic component. Diversity of substrate reduction is provided by the presence of a cleft in the catalytic component that can either accommodate a large cofactor (FeMoco) or a large substrate (protochlorophylide). Although none of the alpha subunit related sequences we have analysed contain the FeMoco ligand His442, it is not possible to distinguish whether the function of these sequences is likely to relate to catalysis (i.e. NifDK-like) or to biosynthesis (i.e. NifEN-like). Biochemical and structural studies of NifEN reveal its functional diversity, since it can catalyse cluster conversion, molybdenum incorporation into the cofactor in association with NifH, and potentially the incorporation of homocitrate into FeMoco [9]. Although the primary role of NifEN is to provide the machinery for FeMoco biosynthesis, it has also been shown to catalyse reduction of some nitrogenase substrates, albeit with relatively low efficiency [13].

Nitrogenase-like sequences could potentially perform analogous roles in association with a NifH-like component. The genomic organisation of these proteins may provide some clues to their possible metabolic functions (Additional file 4: Figure S2). We note that sequences possessing the equivalent of Cys275 in the alpha subunit are commonly associated with O-acetyl homoserine sulfhydrolase or cysteine synthase, suggesting a potential involvement in sulphur metabolism (e.g. *Rhodospirillum rubrum* ATCC 11170, *Clostridium beijerinckii* NCIMB 8052, *Geobacter sp*. FRC-32, Additional file 4: Figure S2). In other cases, nitrogenase–like sequences are co-located with ABC transporter systems (e.g. *Clostridium cellulovorans* 743B, *Methanocorpusculum labreanum* Z, *Clostridium botulinum* A2 Kyoto-F). Possibly this might provide a mechanism for coupling metal transport to the assembly of a metal cofactor. In *Coprococcus catus* GD/7 and other representatives of the Firmicutes, NifHEN-like proteins are associated with hydrogenase maturation proteins and may possibly play a role in the assembly of the active site metallocluster. The NifD proteins present in methanogenic Archaea have been proposed to function in coenzyme F430 biosynthesis, and NflD has been shown to co-purify with a NifH-like gene, NflH [31]. In some cases we observe that NflD homologs are adjacent to NflH and a gene involved in a late step in cobalamin biosynthesis, which encodes cobyrinic acid a,c-diamide synthase (Additional file 4: Figure S2). This may imply that these proteins function in cobalamin reduction.

The NflD single subunit enzymes appear to be the early ancestors of both the bacteriochlorophyll biosynthesis proteins (BchN and BchB) and the nitrogenases (Nif/Vnf/AnfDK) [24,27,31]. Recent evolutionary studies suggest that nitrogen fixation originated after the emergence of bacteriochlorophyll biosynthesis [25] and consequently spread to diverse microbial lineages via lateral gene transfer [24,27]. Potentially, the additional NifDK-like sequences that we have identified may be representative of ancestors that arose after the duplication event that led to the emergence of the alpha and beta subunits of nitrogenase and evolved to perform various metabolic functions. It is important to note that thus far we have only identified nitrogenase-like sequences in obligate or facultative anaerobes, consistent with the view that nitrogenase evolved in anaerobic methanogens and Firmicutes [25]. As noted above these early forms may not have functioned as catalysts, but might have had roles in metallocluster biosynthesis. Although current information on the role of these nitrogenase-like sequences is sparse, future biochemical and structural studies on this hitherto unrecognised group of proteins are likely to provide a rich source of information concerning the evolution and catalytic diversity of these nitrogenase homologs.

## Conclusions

This work led to the identification of 67 potential diazotrophic species included in twelve taxonomic phyla, indicating that this metabolic trait is more widespread than formerly predicted. The identification of a minimum gene set required for nitrogen fixation provides a more robust method for the *in silico* prediction of this biochemical pathway. The occurrence of *nif*-orphan sequences or incomplete gene sets in several species questions single-gene approaches used in phylogenetic studies of nitrogen fixation. Furthermore our analysis highlights the presence of nitrogenase-like sequences with potential to catalyze as-yet unidentified functions.

## Methods

### Survey of nitrogen fixing genes in prokaryotic genomes

Nitrogen fixing genes present in species with completely sequences genomes were identified through the protein database of microbial genomes at the National Center for Biotechnology Information up to July 17^th^ 2011. Only one representative of species containing more than one sequenced genome was manually selected resulting in 999 unique Bacterial species and 93 unique Archaeal species. BLAST [32] searches used as queries the *A. vinelandii* nitrogen fixing protein sequences: NifH (Avin_01380), NifD (Avin_01390), NifK (Avin_01400), NifE (Avin_01450), NifN (Avin_01460), NifU (Avin_01620), NifS (Avin_01630), NifV (Avin_01640), NifB (Avin_51010), NifQ (Avin_51040), AnfG (Avin_48980), and VnfG (Avin_02600). Initially hits were selected based on a relatively weak threshold (≥ 20%amino acid identity over the query length); using the minimum gene set criterion, hits to *anf/vnfG,* and presence of synteny the initial list was refined, yielding the protein sequences listed in Additional file 2: Table S2, Additional file 2: Table S3, Additional file 2: Table S4.

### Selection and phylogenetic analysis of nitrogenase-like sequences

An initial list of 75 NifD/E and NifK/N-like sequences belonging to the PFAM family PF00148 were selected manually from the IMG database [33] (http://img.jgi.doe.gov) and then used as queries in a BLAST [32] search against the NCBI NR protein database with an e-value cut-off of 10^−20^. This returned 1117 unique geneIDs, which were then filtered against known NifD/E and NifK/N sequences (Additional file 2: Table S3) to remove hits to conventional nitrogenase. The remaining 900 unique gene IDs were further filtered with a BLAST search against ChlB (accession GenBank:AAT28195.1), BchB (SwissProt:Q3APL0.1), ChlN (GenBank:AAP99591.1) and BchN (SwissProt:Q3APK9.1) to remove homologs of protochlorophylide reductase. Fused protein sequences (NifHD/E) were also filtered out and were not subject to further phylogenetic analysis. Another filtering was done with a preliminary tree built using FastTree 2.1 [34] to identify very similar sequences; only one member of each set of similar sequences was kept. The final compilation contained 472 unique gene IDs.

Manual inspection of the 472-sequence tree yielded a “core” list of 73 representative sequences. These 73 sequences were then aligned with ClustalW version 2.1 [35] with the Gonnet 250 protein matrix and default pairwise alignment options. A phylogenetic tree was built with FastTree 2.1 [34] using the WAG + gamma20 likelihood model; the result is shown in Figure 4.

## **Competing interests**

The authors declare no competing interests.

## Authors’ contributions

PDS, JCS and RD designed the study. PDS, FZ, RD performed searches and RD, JCS and SWM performed phylogenetic analyses. PDS, FZ, JCS, RD drafted and revised the manuscript. All authors approved the final version of the manuscript for publication.

## Supplementary Material

Additional file 1 **Table S1.** Reference table of known diazotrophs [36-109]. Click here for file

Additional file 2 **Table S2.** Nitrogen fixation genes (locus tags) of known diazotrophs. **Table S3.** Nitrogen fixation genes (locus tags) of potential diazotrophs. **Table S4.** Nitrogen fixation genes (locus tags) of Group-C species. Click here for file

Additional file 3 **Figure S1.** Neighbor joining phylogenetic tree of the Nif/Vnf/AnfD and K sequences derived from the species shown in Figure 3. Click here for file

Additional file 4 **Figure S2.** Gene neighborhoods of selected nitrogenase-like proteins. Click here for file

## References

[B1] Seefeldt LC Hoffman BM Dean DR Mechanism of Mo-dependent nitrogenase Annu Rev Biochem 2009 78 701 722 19489731 10.1146/annurev.biochem.78.070907.103812 PMC2814439

[B2] Hartmann LS Barnum SR Inferring the evolutionary history of Mo-dependent nitrogen fixation from phylogenetic studies of nifK and nifDK J Mol Evol 2010 71 70 85 20640414 10.1007/s00239-010-9365-8

[B3] O’Carroll IP Dos Santos PC Genomic analysis of nitrogen fixation Methods Mol Biol 2011 766 49 65 21833860 10.1007/978-1-61779-194-9_4

[B4] Zehr JP Jenkins BD Short SM Steward GF Nitrogenase gene diversity and microbial community structure: a cross-system comparison Environ Microbiol 2003 5 539 554 12823187 10.1046/j.1462-2920.2003.00451.x

[B5] Ribbe M Gadkari D Meyer O N2 fixation by Streptomyces thermoautotrophicus involves a molybdenum- dinitrogenase and a manganese-superoxide oxidoreductase that couple N2 reduction to the oxidation of superoxide produced from O2 by a molybdenum-CO dehydrogenase J Biol Chem 1997 272 26627 26633 9334244 10.1074/jbc.272.42.26627

[B6] Zehr JP Nitrogen fixation by marine cyanobacteria Trends Microbiol 2011 19 162 173 21227699 10.1016/j.tim.2010.12.004

[B7] Stark M Berger SA Stamatakis A von Mering C MLTreeMap - accurate Maximum Likelihood placement of environmental DNA sequences into taxonomic and functional reference phylogenies BMC Genomics 2010 11 461 20687950 10.1186/1471-2164-11-461 PMC3091657

[B8] Turk KA Rees AP Zehr JP Pereira N Swift P Shelley R Lohan M Woodward EM Gilbert J Nitrogen fixation and nitrogenase (nifH) expression in tropical waters of the eastern North Atlantic ISME J 2011 5 1201 1212 21228888 10.1038/ismej.2010.205 PMC3146282

[B9] Rubio LM Ludden PW Biosynthesis of the iron-molybdenum cofactor of nitrogenase Annu Rev Microbiol 2008 62 93 111 18429691 10.1146/annurev.micro.62.081307.162737

[B10] Hamilton TL Ludwig M Dixon R Boyd ES Dos Santos PC Setubal JC Bryant DA Dean DR Peters JW Transcriptional profiling of nitrogen fixation in Azotobacter vinelandii J Bacteriol 2011 193 4477 4486 21724999 10.1128/JB.05099-11 PMC3165507

[B11] Yan Y Ping S Peng J Han Y Li L Yang J Dou Y Li Y Fan H Fan Y Global transcriptional analysis of nitrogen fixation and ammonium repression in root-associated Pseudomonas stutzeri A1501 BMC Genomics 2011 11 11 20053297 10.1186/1471-2164-11-11 PMC2820453

[B12] Hu Y Ribbe MW Biosynthesis of Nitrogenase FeMoco Coord Chem Rev 2011 255 1218 1224 21503270 10.1016/j.ccr.2010.11.018 PMC3077758

[B13] Kaiser JT Hu Y Wiig JA Rees DC Ribbe MW Structure of precursor-bound NifEN: a nitrogenase FeMo cofactor maturase/insertase Science 2011 331 91 94 21212358 10.1126/science.1196954 PMC3138709

[B14] Brigle KE Weiss CM Newton WE Dean DR Products of the iron-molybdenum cofactor-specific biosynthetic genes, nifE and nifN, are structurally homologous to the products of the nitrogenase molybdenum-iron protein genes, nifH and nifK J Bacteriol 1987 169 1547 1553 3470285 10.1128/jb.169.4.1547-1553.1987 PMC211981

[B15] Benson DA Karsch-Mizrachi I Lipman DJ Ostell J Sayers EW GenBank Nucleic Acids Res 2011 39 D32 D37 21071399 10.1093/nar/gkq1079 PMC3013681

[B16] Yagi JM Sims D Brettin T Bruce D Madsen EL The genome of Polaromonas naphthalenivorans strain CJ2, isolated from coal tar-contaminated sediment, reveals physiological and metabolic versatility and evolution through extensive horizontal gene transfer Environ Microbiol 2009 11 2253 2270 19453698 10.1111/j.1462-2920.2009.01947.x

[B17] Lee PK He J Zinder SH Alvarez-Cohen L Evidence for nitrogen fixation by “Dehalococcoides ethenogenes” strain 195 Appl Environ Microbiol 2009 75 7551 7555 19820162 10.1128/AEM.01886-09 PMC2786412

[B18] Martinez-Aguilar L Diaz R Pena-Cabriales JJ Estrada-de Los Santos P Dunn MF Caballero-Mellado J Multichromosomal genome structure and confirmation of diazotrophy in novel plant-associated Burkholderia species Appl Environ Microbiol 2008 74 4574 4579 18502926 10.1128/AEM.00201-08 PMC2493167

[B19] Larsson J Nylander JA Bergman B Genome fluctuations in cyanobacteria reflect evolutionary, developmental and adaptive traits BMC Evol Biol 2011 11 187 21718514 10.1186/1471-2148-11-187 PMC3141437

[B20] Masson-Boivin C Giraud E Perret X Batut J Establishing nitrogen-fixing symbiosis with legumes: how many rhizobium recipes? Trends Microbiol 2009 17 458 466 19766492 10.1016/j.tim.2009.07.004

[B21] Eady RR Structure-function-relationships of alternative nitrogenases Chem Rev 1996 96 3013 3030 11848850 10.1021/cr950057h

[B22] Mehta MP Baross JA Nitrogen fixation at 92 degrees C by a hydrothermal vent archaeon Science 2006 314 1783 1786 17170307 10.1126/science.1134772

[B23] Kechris KJ Lin JC Bickel PJ Glazer AN Quantitative exploration of the occurrence of lateral gene transfer by using nitrogen fixation genes as a case study Proc Natl Acad Sci U S A 2006 103 9584 9589 16769896 10.1073/pnas.0603534103 PMC1480450

[B24] Boyd ES Hamilton TL Peters JW An alternative path for the evolution of biological nitrogen fixation Frontiers in Microbiology 2011 2 205 22065963 10.3389/fmicb.2011.00205 PMC3207485

[B25] Boyd ES Anbar AD Miller S Hamilton TL Lavin M Peters JW A late methanogen origin for molybdenum-dependent nitrogenase Geobiology 2011 9 221 232 21504537 10.1111/j.1472-4669.2011.00278.x

[B26] Muraki N Nomata J Ebata K Mizoguchi T Shiba T Tamiaki H Kurisu G Fujita Y X-ray crystal structure of the light-independent protochlorophyllide reductase Nature 2010 465 110 114 20400946 10.1038/nature08950

[B27] Raymond J Siefert JL Staples CR Blankenship RE The natural history of nitrogen fixation Mol Biol Evol 2004 21 541 554 14694078 10.1093/molbev/msh047

[B28] Hu Y Yoshizawa JM Fay AW Lee CC Wiig JA Ribbe MW Catalytic activities of NifEN: implications for nitrogenase evolution and mechanism Proc Natl Acad Sci U S A 2009 106 16962 16966 19805110 10.1073/pnas.0907872106 PMC2761346

[B29] Overbeek R Begley T Butler RM Choudhuri JV Chuang HY Cohoon M de Crecy-Lagard V Diaz N Disz T Edwards R The subsystems approach to genome annotation and its use in the project to annotate 1000 genomes Nucleic Acids Res 2005 33 5691 5702 16214803 10.1093/nar/gki866 PMC1251668

[B30] Soboh B Boyd ES Zhao D Peters JW Rubio LM Substrate specificity and evolutionary implications of a NifDK enzyme carrying NifB-co at its active site FEBS Lett 2010 584 1487 1492 20219465 10.1016/j.febslet.2010.02.064

[B31] Staples CR Lahiri S Raymond J Von Herbulis L Mukhophadhyay B Blankenship RE Expression and association of group IV nitrogenase NifD and NifH homologs in the non-nitrogen-fixing archaeon Methanocaldococcus jannaschii J Bacteriol 2007 189 7392 7398 17660283 10.1128/JB.00876-07 PMC2168459

[B32] Altschul SF Madden TL Schaffer AA Zhang J Zhang Z Miller W Lipman DJ Gapped BLAST and PSI-BLAST: a new generation of protein database search programs Nucleic Acids Res 1997 25 3389 3402 9254694 10.1093/nar/25.17.3389 PMC146917

[B33] Markowitz VM Chen IM Palaniappan K Chu K Szeto E Grechkin Y Ratner A Anderson I Lykidis A Mavromatis K The integrated microbial genomes system: an expanding comparative analysis resource Nucleic Acids Res 2010 38 D382 D390 19864254 10.1093/nar/gkp887 PMC2808961

[B34] Price MN Dehal PS Arkin AP FastTree 2–approximately maximum-likelihood trees for large alignments PLoS One 2010 5 e9490 20224823 10.1371/journal.pone.0009490 PMC2835736

[B35] Larkin MA Blackshields G Brown NP Chenna R McGettigan PA McWilliam H Valentin F Wallace IM Wilm A Lopez R Clustal W and Clustal X version 2.0 Bioinformatics 2007 23 2947 2948 17846036 10.1093/bioinformatics/btm404

[B36] Mackintosh ME Nitrogen-fixation by Thiobacillus-Ferrooxidans J Gen Microbiol 1978 105 215 218

[B37] Dincturk HB Demir V Rnf Genes in purple sulfur bacterium Allochromatium vinosum Turk J Biol 2006 30 143 147

[B38] Berman-Frank I Lundgren P Falkowski P Nitrogen fixation and photosynthetic oxygen evolution in cyanobacteria Res Microbiol 2003 154 157 164 12706503 10.1016/S0923-2508(03)00029-9

[B39] McClung CR Patriquin DG Davis RE Campylobacter nitrofigilis sp. nov., a Nitrogen-Fixing Bacterium Associated with Roots of Spartina alterniflora Loisel International Journal of Systematic and Evolutionary Microbiology 1983 33 605 613

[B40] Reinhold-Hurek B Hurek T Gillis M Hoste B Kersters K Deley J Diazotrophs repeatedly isolated from roots of kallar grass form a new genus, Azoarcus Nitrogen Fixation: Achievements and Objectives; 8th International Congress on Nitrogen Fixation 1990 43216534933

[B41] Dreyfus B Garcia JL Gillis M Characterization of Azorhizobium-Caulinodans Gen-Nov, Sp-Nov, a Stem-Nodulating Nitrogen-Fixing Bacterium Isolated from Sesbania-Rostrata Int J Syst Bacteriol 1988 38 89 98

[B42] Kaneko T Minamisawa K Isawa T Nakatsukasa H Mitsui H Kawaharada Y Nakamura Y Watanabe A Kawashima K Ono A Complete genomic structure of the cultivated rice endophyte Azospirillum sp. B510 DNA Res 2010 17 37 50 20047946 10.1093/dnares/dsp026 PMC2818188

[B43] Setubal JC dos Santos P Goldman BS Ertesvag H Espin G Rubio LM Valla S Almeida NF Balasubramanian D Cromes L Genome sequence of Azotobacter vinelandii, an obligate aerobe specialized to support diverse anaerobic metabolic processes J Bacteriol 2009 191 4534 4545 19429624 10.1128/JB.00504-09 PMC2704721

[B44] Kennedy C Rudnick P MacDonald T Melton T Garrity GM Genus Azotobacter Bergey's manual of sytematic bacteriology 2005 New York, NY: Springer-Verlag 2(B): 384-401

[B45] Molouba F Lorquin J Willems A Hoste B Giraud E Dreyfus B Gillis M de Lajudie P Masson-Boivin C Photosynthetic bradyrhizobia from Aeschynomene spp. are specific to stem-nodulated species and form a separate 16 S ribosomal DNA restriction fragment length polymorphism group Appl Environ Microbiol 1999 65 3084 3094 10388707 10.1128/aem.65.7.3084-3094.1999 PMC91460

[B46] Elliott GN Chen WM Chou JH Wang HC Sheu SY Perin L Reis VM Moulin L Simon MF Burkholderia phymatum is a highly effective nitrogen-fixing symbiont of Mimosa spp. and fixes nitrogen ex planta New Phytol 2007 173 168 180 17176403 10.1111/j.1469-8137.2006.01894.x

[B47] Boddey RM Urquiaga S Alves BJR Reis V Endophytic nitrogen fixation in sugarcane: present knowledge and future applications Plant Soil 2003 252 139 149

[B48] Van VT Berge O Balandreau J Ke SN Heulin T Isolation and nitrogenase activity of Burkholderia vietnamiensis, a nitrogen-fixing bacterium associated with rice (Oryza sativa L) on a sulphate acid soil of Vietnam Agronomie 1996 16 479 491

[B49] Caballero-Mellado J Martinez-Aguilar L Diaz R Pena-Cabriales JJ Estrada-de los Santos P Dunn MF Multichromosomal genome structure and confirmation of diazotrophy in novel plant-associated Burkholderia species Applied and Environmental Microbiology 2008 74 4574 4579 18502926 10.1128/AEM.00201-08 PMC2493167

[B50] Postgate JR Cannon FC The molecular and genetic manipulation of nitrogen-fixation Philos Trans R Soc Lond B Biol Sci 1981 292 589 599

[B51] Postgate JR Gibson AH, Newton WE Microbiology of the free-living nitrogen fixing bacteria, excluding cyanobacteria Current Perspectives in Nitrogen Fixation 1981 Canberra: Australian Academy of Science 217 228

[B52] Wahlund TM Madigan MT Nitrogen-fixation by the thermophilic green sulfur bacterium chlorobium-tepidum J Bacteriol 1993 175 474 478 8093448 10.1128/jb.175.2.474-478.1993 PMC196162

[B53] Chen JS Toth J Kasap M Nitrogen-fixation genes and nitrogenase activity in Clostridium acetobutylicum and Clostridium beijerinckii J Ind Microbiol Biotechnol 2001 27 281 286 11781802 10.1038/sj.jim.7000083

[B54] Kanamori K Weiss RL Roberts JD Ammonia assimilation pathways in nitrogen-fixing clostridium-kluyverii and clostridium-butyricum J Bacteriol 1989 171 2148 2154 2564848 10.1128/jb.171.4.2148-2154.1989 PMC209870

[B55] Masson-Boivin C Amadou C Pascal G Mangenot S Glew M Bontemps C Capela D Carrere S Cruveiller S Dossat C Genome sequence of the beta-rhizobium Cupriavidus taiwanensis and comparative genomics of rhizobia Genome Res 2008 18 1472 1483 18490699 10.1101/gr.076448.108 PMC2527706

[B56] Zehr JP Bench SR Carter BJ Hewson I Niazi F Shi T Tripp HJ Affourtit JP Globally Distributed Uncultivated Oceanic N(2)-Fixing Cyanobacteria Lack Oxygenic Photosystem II Science 2008 322 1110 1112 19008448 10.1126/science.1165340

[B57] Pakrasi HB Welsh EA Liberton M Stoeckel J Loh T Elvitigala T Wang C Wollam A Fulton RS Clifton SW The genome of Cyanothece 51142, a unicellular diazotrophic cyanobacterium important in the marine nitrogen cycle Proc Natl Acad Sci U S A 2008 105 15094 15099 18812508 10.1073/pnas.0805418105 PMC2567498

[B58] Alvarez-Cohen L Lee PKH He JZ Zinder SH Evidence for Nitrogen Fixation by “Dehalococcoides ethenogenes” Strain 195 Appl Environ Microbiol 2009 75 7551 7555 19820162 10.1128/AEM.01886-09 PMC2786412

[B59] Postgate JR Biochemical and physiological studies with free-living, nitrogen-fixing bacteria Plant Soil 1971 35 551 559

[B60] Kim SH Harzman C Davis JK Hutcheson R Broderick JB Marsh TL Tiedje JM Genome sequence of Desulfitobacterium hafniense DCB-2, a Gram-positive anaerobe capable of dehalogenation and metal reduction BMC Microbiol 2012 12 21 22316246 10.1186/1471-2180-12-21 PMC3306737

[B61] Riederer-Henderson MA Wilson PW Nitrogen fixation by sulphate-reducing bacteria J Gen Microbiol 1970 61 27 31 5489063 10.1099/00221287-61-1-27

[B62] Harriott OT Hosted TJ Benson DR Sequences of nifX, nifW, nifZ, nifB and two ORF in the Frankia nitrogen fixation gene cluster Gene 1995 161 63 67 7642138 10.1016/0378-1119(95)00300-u

[B63] Ligon JM Nakas JP Isolation and Characterization of Frankia sp. Strain FaC1 Genes Involved in Nitrogen Fixation Appl Environ Microbiol 1987 53 2321 2327 16347453 10.1128/aem.53.10.2321-2327.1987 PMC204107

[B64] Mouser PJ N’Guessan AL Elifantz H Holmes DE Williams KH Wilkins MJ Long PE Lovley DR Influence of heterogeneous ammonium availability on bacterial community structure and the expression of nitrogen fixation and ammonium transporter genes during in situ bioremediation of uranium-contaminated groundwater Environ Sci Technol 2009 43 4386 4392 19603651 10.1021/es8031055

[B65] Bazylinski DA Dean AJ Schuler D Phillips EJ Lovley DR N2-dependent growth and nitrogenase activity in the metal-metabolizing bacteria, Geobacter and Magnetospirillum species Environ Microbiol 2000 2 266 273 11200427 10.1046/j.1462-2920.2000.00096.x

[B66] Methe BA Nelson KE Eisen JA Paulsen IT Nelson W Heidelberg JF Wu D Wu M Ward N Beanan MJ Genome of Geobacter sulfurreducens: Metal reduction in subsurface environments Science 2003 302 1967 1969 14671304 10.1126/science.1088727

[B67] Ureta A Nordlund S Evidence for conformational protection of nitrogenase against oxygen in Gluconacetobacter diazotrophicus by a putative FeSII protein J Bacteriol 2002 184 5805 5809 12270840 10.1128/JB.184.20.5805-5809.2002 PMC139593

[B68] Tsuihiji H Yamazaki Y Kamikubo H Imamoto Y Kataoka M Cloning and characterization of nif structural and regulatory genes in the purple sulfur bacterium, Halorhodospira halophila J Biosci Bioeng 2006 101 263 270 16716929 10.1263/jbb.101.263

[B69] Sattley WM Madigan MT Swingley WD Cheung PC Clocksin KM Conrad AL Dejesa LC Honchak BM Jung DO Karbach LE The genome of Heliobacterium modesticaldum, a phototrophic representative of the Firmicutes containing the simplest photosynthetic apparatus J Bacteriol 2008 190 4687 4696 18441057 10.1128/JB.00299-08 PMC2446807

[B70] Noindorf L Bonatto AC Monteiro RA Souza EM Rigo LU Pedrosa FO Steffens MB Chubatsu LS Role of PII proteins in nitrogen fixation control of Herbaspirillum seropedicae strain SmR1 BMC Microbiol 2011 11 8 21223584 10.1186/1471-2180-11-8 PMC3023670

[B71] Fouts DE Tyler HL Deboy RT Daugherty S Ren QH Badger JH Durkin AS Huot H Shrivastava S Kothari S Complete Genome Sequence of the N(2)-Fixing Broad Host Range Endophyte Klebsiella pneumoniae 342 and Virulence Predictions Verified in Mice Plos Genetics 2008 4 e1000141 18654632 10.1371/journal.pgen.1000141 PMC2453333

[B72] Pinto-Tomas AA Anderson MA Suen G Stevenson DM Chu FS Cleland WW Weimer PJ Currie CR Symbiotic nitrogen fixation in the fungus gardens of leaf-cutter ants Science 2009 326 1120 1123 19965433 10.1126/science.1173036

[B73] Nandasena KG O’Hara GW Tiwari RP Sezmis E Howieson JG In situ lateral transfer of symbiosis islands results in rapid evolution of diverse competitive strains of mesorhizobia suboptimal in symbiotic nitrogen fixation on the pasture legume Biserrula pelecinus L Environ Microbiol 2007 9 2496 2511 17803775 10.1111/j.1462-2920.2007.01368.x

[B74] Kaneko T Nakamura Y Sato S Asamizu E Kato T Sasamoto S Watanabe A Idesawa K Ishikawa A Kawashima K Complete genome structure of the nitrogen-fixing symbiotic bacterium Mesorhizobium loti (supplement) DNA Res 2000 7 381 406 11214974 10.1093/dnares/7.6.381

[B75] Dudeja NPS SS Poonam Sharma Gupta SC Ramesh Chandra Bansi Dhar Bansal RK Brahmaprakash GP Potdukhe SR Gundappagol RC Biofertilizer Technology and Pulse Production Bioaugmentation, Biostimulation and Biocontrol Soil Biology 2011 28 43 63

[B76] Pine L Haas V Barker HA Metabolism of glucose by Butyribacterium rettgeri J Bacteriol 1954 68 227 230 13183933 10.1128/jb.68.2.227-230.1954 PMC357370

[B77] Kendall MM Liu Y Sieprawska-Lupa M Stetter KO Whitman WB Boone DR Methanococcus aeolicus sp nov., a mesophilic, methanogenic archaeon from shallow and deep marine sediments Int J Syst Evol Microbiol 2006 56 1525 1529 16825624 10.1099/ijs.0.64216-0

[B78] Leigh JA Nitrogen fixation in methanogens: the archaeal perspective Curr Issues Mol Biol 2000 2 125 131 11471757

[B79] Boccazzi P Zhang JK Metcalf WW Generation of dominant selectable markers for resistance to pseudomonic acid by cloning and mutagenesis of the ileS gene from the archaeon Methanosarcina barkeri fusaro J Bacteriol 2000 182 2611 2618 10762266 10.1128/jb.182.9.2611-2618.2000 PMC111328

[B80] Lobo AL Zinder SH Diazotrophy and Nitrogenase Activity in the Archaebacterium Methanosarcina-Barkeri 227 Appl Environ Microbiol 1988 54 1656 1661 16347675 10.1128/aem.54.7.1656-1661.1988 PMC202723

[B81] Ehlers C Veit K Gottschalk G Schmitz RA Functional organization of a single nif cluster in the mesophilic archaeon Methanosarcina mazei strain Go1 Archaea 2002 1 143 150 15803652 10.1155/2002/362813 PMC2685556

[B82] Fardeau ML Peillex JP Belaich JP Energetics of the Growth of Methanobacterium-Thermoautotrophicum and Methanococcus-Thermolithotrophicus on Ammonium-Chloride and Dinitrogen Arch Microbiol 1987 148 128 131

[B83] Jourand P Giraud E Bena G Sy A Willems A Gillis M Dreyfus B de Lajudie P Methylobacterium nodulans sp. nov., for a group of aerobic, facultatively methylotrophic, legume root-nodule-forming and nitrogen-fixing bacteria Int J Syst Evol Microbiol 2004 54 2269 2273 15545469 10.1099/ijs.0.02902-0

[B84] Dunfield PF Khmelenina VN Suzina NE Trotsenko YA Dedysh SN Methylocella silvestris sp. nov., a novel methanotroph isolated from an acidic forest cambisol Int J Syst Evol Microbiol 2003 53 1231 1239 13130000 10.1099/ijs.0.02481-0

[B85] Murrell JC Dalton H Nitrogen-Fixation in Obligate Methanotrophs J Gen Microbiol 1983 129 3481 3486

[B86] Romanovskaia VA Shurova ZP Iurchenko VV Tkachuk LV Malashenko Iu R [Ability of obligate methylotrophs to perform nitrogen fixation] Mikrobiologiia 1977 46 66 70 404513

[B87] Vaishampayan A Sinha RP Gupta AK Hader DP A cyanobacterial recombination study, involving an efficient N2-fixing non-heterocystous partner Microbiol Res 2000 155 137 141 11061181 10.1016/S0944-5013(00)80026-9

[B88] Meeks JC Campbell EL Summers ML Wong FC Cellular differentiation in the cyanobacterium Nostoc punctiforme Arch Microbiol 2002 178 395 403 12420158 10.1007/s00203-002-0476-5

[B89] Xu XD Zhang W Du Y Khudyakov I Fan Q Gao H Ning DG Wolk CP A gene cluster that regulates both heterocyst differentiation and pattern formation in Anabaena sp strain PCC 7120 Mol Microbiol 2007 66 1429 1443 18045384 10.1111/j.1365-2958.2007.05997.x

[B90] Loiret FG Grimm B Hajirezaei MR Kleiner D Ortega E Inoculation of sugarcane with Pantoea sp. increases amino acid contents in shoot tissues; serine, alanine, glutamine and asparagine permit concomitantly ammonium excretion and nitrogenase activity of the bacterium J Plant Physiol 2009 166 1152 1161 19215998 10.1016/j.jplph.2009.01.002

[B91] Hansen TA Nienhuiskuiper HE Stams AJM A Rod-Shaped, Gram-Negative, Propionigenic Bacterium with a Wide Substrate Range and the Ability to Fix Molecular Nitrogen Arch Microbiol 1990 155 42 45

[B92] Madsen EL Yagi JM Sims D Brettin T Bruce D The genome of Polaromonas naphthalenivorans strain CJ2, isolated from coal tar-contaminated sediment, reveals physiological and metabolic versatility and evolution through extensive horizontal gene transfer Environ Microbiol 2009 11 2253 2270 19453698 10.1111/j.1462-2920.2009.01947.x

[B93] Yan Y Yang J Dou Y Chen M Ping S Peng J Lu W Zhang W Yao Z Li H Nitrogen fixation island and rhizosphere competence traits in the genome of root-associated Pseudomonas stutzeri A1501 Proc Natl Acad Sci U S A 2008 105 7564 7569 18495935 10.1073/pnas.0801093105 PMC2396677

[B94] Gonzalez V Santamaria RI Bustos P Hernandez-Gonzalez I Medrano-Soto A Moreno-Hagelsieb G Janga SC Ramirez MA Jimenez-Jacinto V Collado-Vides J Davila G The partitioned Rhizobium etli genome: genetic and metabolic redundancy in seven interacting replicons Proc Natl Acad Sci U S A 2006 103 3834 3839 16505379 10.1073/pnas.0508502103 PMC1383491

[B95] Finnie C Maeda K Ostergaard O Svensson B Identification, cloning and characterization of two thioredoxin H isoforms, HvTrxh1 and HvTrxh2, from the barley seed proteome Eur J Biochem 2003 270 2633 2643 12787030 10.1046/j.1432-1033.2003.03637.x

[B96] Young JPW Crossman LC Johnston AWB Thomson NR Ghazoui ZF Hull KH Wexler M Curson ARJ Todd JD Poole PS The genome of Rhizobium leguminosarum has recognizable core and accessory components Genome Biology 2006 7 R34 16640791 10.1186/gb-2006-7-4-r34 PMC1557990

[B97] Djordjevic SP Chen H Batley M Redmond JW Rolfe BG Nitrogen-Fixation Ability of Exopolysaccharide Synthesis Mutants of Rhizobium Sp Strain Ngr234 and Rhizobium-Trifolii Is Restored by the Addition of Homologous Exopolysaccharides J Bacteriol 1987 169 53 60 3025187 10.1128/jb.169.1.53-60.1987 PMC211732

[B98] Haselkorn R Strnad H Lapidus A Paces J Ulbrich P Vlcek C Paces V Complete genome sequence of the photosynthetic purple nonsulfur bacterium rhodobacter capsulatus SB 1003 J Bacteriol 2010 192 3545 3546 20418398 10.1128/JB.00366-10 PMC2897665

[B99] Whittenbury R Dow CS Morphogenesis and differentiation in rhodomicrobium-vannielii and other budding and prosthecate bacteria Bacteriol Rev 1977 41 754 808 334156 10.1128/br.41.3.754-808.1977 PMC414022

[B100] Larimer FW Chain P Hauser L Lamerdin J Malfatti S Do L Land ML Pelletier DA Beatty JT Lang AS Complete genome sequence of the metabolically versatile photosynthetic bacterium Rhodopseudomonas palustris Nat Biotechnol 2004 22 55 61 14704707 10.1038/nbt923

[B101] Lu YK Marden J Han M Swingley WD Mastrian SD Chowdhury SR Hao J Helmy T Kim S Kurdoglu AA Metabolic flexibility revealed in the genome of the cyst-forming alpha-1 proteobacterium Rhodospirillum centenum BMC Genomics 2010 11 325 20500872 10.1186/1471-2164-11-325 PMC2890560

[B102] Reslewic S Zhou S Place M Zhang Y Briska A Goldstein S Churas C Runnheim R Forrest D Lim A Whole-genome shotgun optical mapping of Rhodospirillum rubrum Appl Environ Microbiol 2005 71 5511 5522 16151144 10.1128/AEM.71.9.5511-5522.2005 PMC1214604

[B103] Krishnan HB Jiang GQ Krishnan AH Kim YW Wacek TJ A functional myo-inositol dehydrogenase gene is required for efficient nitrogen fixation and competitiveness of Sinorhizobium fredii USDA191 to nodulate soybean (Glycine max [L.] Merr.) J Bacteriol 2001 183 2595 2604 11274120 10.1128/JB.183.8.2595-2604.2001 PMC95177

[B104] Terpolilli JJ O’Hara GW Tiwari RP Dilworth MJ Howieson JG The model legume Medicago truncatula A17 is poorly matched for N2 fixation with the sequenced microsymbiont Sinorhizobium meliloti 1021 New Phytol 2008 179 62 66 18422896 10.1111/j.1469-8137.2008.02464.x

[B105] Galibert F Finan TM Long SR Puhler A Abola P Ampe F Barloy-Hubler F Barnett MJ Becker A Boistard P The composite genome of the legume symbiont Sinorhizobium meliloti Science 2001 293 668 672 11474104 10.1126/science.1060966

[B106] Steunou AS Jensen SI Brecht E Becraft ED Bateson MM Kilian O Bhaya D Ward DM Peters JW Grossman AR Kuhl M Regulation of nif gene expression and the energetics of N2 fixation over the diel cycle in a hot spring microbial mat ISME J 2008 2 364 378 18323780 10.1038/ismej.2007.117

[B107] Distel DL Morrill W MacLaren-Toussaint N Franks D Waterbury J Teredinibacter turnerae gen. nov., sp. nov., a dinitrogen-fixing, cellulolytic, endosymbiotic gamma-proteobacterium isolated from the gills of wood-boring molluscs (Bivalvia: Teredinidae) Int J Syst Evol Microbiol 2002 52 2261 2269 12508896 10.1099/00207713-52-6-2261

[B108] Ramamurthy VD Krishnamurthy S Nitrogen-fixation by the blue-green alga, Trichodesmium erythraeum (Ehr.) Curr Sci 1968 37 21 22

[B109] Schneider K Muller A Krahn E Hagen WR Wassink H Knuttel KH The molybdenum nitrogenase from wild-type Xanthobacter autotrophicus exhibits properties reminiscent of alternative nitrogenases Eur J Biochem 1995 230 666 675 7607241 10.1111/j.1432-1033.1995.0666h.x

