# Supplementary material for: Distribution of nitrogen fixation and nitrogenase-like sequences amongst microbial genomes
Source: BMC Genomics. 2012 May 3;13:162. doi: 10.1186/1471-2164-13-162 (PMC3464626; doi:10.1186/1471-2164-13-162)
Supplement: Additional file 1 — Table S1. Reference table of known diazotrophs [36-109]. [file 1471-2164-13-162-S1.doc]

**Table S1**

| **Known diazotroph** | **Reference** |
| --- | --- |
| Acidithiobacillus ferrooxidans ATCC 23270 | [36] |
| Allochromatium vinosum DSM 180 | [37] |
| Anabaena variabilis ATCC 29413 | [38] |
| Arcobacter nitrofigilis DSM 7299 | [39] |
| Azoarcus sp. BH72 | [40] |
| Azorhizobium caulinodans ORS 571 | [41] |
| Azospirillum sp. B510 | [42] |
| Azotobacter vinelandii AvOP | [43] |
| Beijerinckia indica indica ATCC 9039 | [44] |
| Bradyrhizobium japonicum USDA 110 | [44] |
| Bradyrhizobium sp. BTAi1 | [45] |
| Burkholderia phymatum STM815 | [46] |
| Burkholderia sp. CCGE1002 | [47] |
| Burkholderia vietnamiensis G4 | [48] |
| Burkholderia xenovorans LB400 | [49] |
| Chlorobaculum parvum NCIB 8327 | [50] |
| Chlorobium limicola DSM 245 | [51] |
| Chlorobium phaeobacteroides BS1 | [51] |
| Chlorobium tepidum TLS | [52] |
| Clostridium acetobutylicum ATCC 824 | [53] |
| Clostridium beijerinckii NCIMB 8052 | [53] |
| Clostridium kluyveri DSM 555 | [54] |
| Cupriavidus taiwanensis | [55] |
| cyanobacterium UCYN-A | [56] |
| Cyanothece sp. ATCC 51142 | [57] |
| Dehalococcoides ethenogenes 195 | [58] |
| Desulfotomaculum ruminis DSM 2154 | [59] |
| Desulfitobacterium hafniense DCB-2 | [60] |
| Desulfovibrio vulgaris vulgaris DP4 | [61] |
| Frankia alni ACN14a | [62] |
| Frankia sp. CcI3 | [63] |
| Geobacter lovleyi SZ | [64] |
| Geobacter metallireducens GS-15 | [65] |
| Geobacter sulfurreducens PCA | [66] |
| Geobacter uraniireducens Rf4 | [64] |
| Gluconacetobacter diazotrophicus PAl 5 | [67] |
| Halorhodospira halophila SL1 | [68] |
| Heliobacterium modesticaldum Ice1 | [69] |
| Herbaspirillum seropedicae SmR1 | [70] |
| Klebsiella pneumoniae 342 | [71] |
| Klebsiella variicola At-22 | [72] |
| Magnetospirillum magneticum AMB-1 | [69] |
| Mesorhizobium ciceri biovar biserrulae WSM1271 | [73] |
| Mesorhizobium loti MAFF303099 | [74] |
| Mesorhizobium opportunistum WSM2075 | [75] |
| Methanobacterium sp. AL-21 | [76] |
| Methanococcus aeolicus Nankai-3 | [77] |
| Methanococcus maripaludis C5 | [78] |
| Methanosarcina acetivorans C2A | [79] |
| Methanosarcina barkeri fusaro | [80] |
| Methanosarcina mazei Go1 | [81] |
| Methanothermobacter thermautotrophicus Delta H | [82] |
| Methylobacterium nodulans ORS 2060 | [83] |
| Methylobacterium sp. 4-46 | [83] |
| Methylocella silvestris BL2 | [84] |
| Methylococcus capsulatus Bath | [85] |
| Methylomonas methanica MC09 | [86] |
| Nostoc azollae 0708 | [87] |
| Nostoc punctiforme PCC 73102 | [88] |
| Nostoc sp. PCC 7120 | [89] |
| Pantoea sp. At-9b | [90] |
| Pelobacter propionicus DSM 2379 | [91] |
| Polaromonas naphthalenivorans CJ2 | [92] |
| Prosthecochloris aestuarii DSM 271 | [50] |
| Pseudomonas stutzeri A1501 | [93] |
| Rhizobium etli CFN 42 | [94] |
| Rhizobium leguminosarum bv. trifolii WSM1325 | [95] |
| Rhizobium leguminosarum bv. viciae 3841 | [96] |
| Rhizobium sp. NGR234 (ANU265) | [97] |
| Rhodobacter capsulatus SB1003 | [98] |
| Rhodobacter sphaeroides ATCC 17029 | [98] |
| Rhodomicrobium vannielii ATCC 17100 | [99] |
| Rhodopseudomonas palustris CGA009 | [100] |
| Rhodospirillum centenum SW | [101] |
| Rhodospirillum rubrum ATCC 11170 | [102] |
| Sinorhizobium fredii NGR234 | [103] |
| Sinorhizobium medicae WSM419 | [104] |
| Sinorhizobium meliloti 1021 | [105] |
| Synechococcus sp. JA-2-3Ba(2-13) | [106] |
| Teredinibacter turnerae T7901 | [107] |
| Trichodesmium erythraeum IMS101 | [108] |
| Xanthobacter autotrophicus Py2 | [109] |
|  |  |
