# Supplementary material for: Distribution of nitrogen fixation and nitrogenase-like sequences amongst microbial genomes
Source: BMC Genomics. 2012 May 3;13:162. doi: 10.1186/1471-2164-13-162 (PMC3464626; doi:10.1186/1471-2164-13-162)
Supplement: Additional file 2 — Table S2. Nitrogen fixation genes (locus tags) of known diazotrophs. Table S3. Nitrogen fixation genes (locus tags) of potential diazotrophs. Table S4. Nitrogen fixation genes (locus tags) of Group-C species. [file 1471-2164-13-162-S2.doc]

| **Known diazotrophs** | **Nitrogen fixation genes (locus tag)** | | | | | | |
| --- | --- | --- | --- | --- | --- | --- | --- |
|  | **NifH** | **NifD** | **NifE** | **NifK** | **NifN** | **NifB** | **Anf/VnfG** |
| Acidithiobacillus ferrooxidans ATCC 23270 | AFE_1522 | AFE_1521 | AFE_1517 | AFE_1520 | AFE_1516 | AFE_1529 |  |
| Allochromatium vinosum DSM 180 | Alvin_1881 | Alvin_1880 | Alvin_0903 | Alvin_1879 | Alvin_0904 | Alvin_3039 |  |
| Anabaena variabilis ATCC 29413 | Ava_3916 | Ava_3917 | Ava_3932 | Ava_3930 | Ava_3933 | Ava_3912 | Ava_4026 |
|  | Ava_4247 | Ava_4248 | Ava_4251 | Ava_4249 | Ava_4251 |  |  |
| Arcobacter nitrofigilis DSM 7299 | Arnit_0049 | Arnit_0047 | Arnit_0073 | Arnit_0046 | Arnit_0068 | Arnit_0080 |  |
| Azoarcus sp. BH72 | azo0538 | azo0539 | azo0562 | azo0540 | azo0561 | azo0523 |  |
| Azorhizobium caulinodans ORS 571 | AZC_1041 | AZC_1040 | AZC_1038 | AZC_1037 | AZC_1039 | AZC_3414 |  |
| Azospirillum sp. B510 | AZL_007710 | AZL_007700 | AZL_007660 | AZL_007690 | AZL_007650 | AZL_022470 |  |
|  |  |  |  |  |  |  |  |
| Azotobacter vinelandii AvOP | Avin_01380 | Avin_01390 | Avin_01450 | Avin_01400 | Avin_01470 | Avin_51010 | Avin_48980 |
|  | Avin_02660 | Avin_02610 | Avin_02770 | Avin_02590 | Avin_02750 |  | Avin_02600 |
|  | Avin_49000 | Avin_48990 |  | Avin_48970 |  |  |  |
| Beijerinckia indica indica ATCC 9039 | Bind_0473 | Bind_0474 | Bind_0476 | Bind_0475 | Bind_0477 | Bind_0396 |  |
| Bradyrhizobium japonicum USDA 110 | blr1769 | blr1743 | blr1745 | blr1744 | blr1746 | blr1759 |  |
| Bradyrhizobium sp. BTAi1 | BBta_5925 | BBta_5924 | BBta_5922 | BBta_5921 | BBta_5923 | BBta_5904 |  |
| Burkholderia phymatum STM815 | Bphy_7753 | Bphy_7754 | Bphy_7729 | Bphy_7730 | Bphy_7755 | Bphy_7742 |  |
|  | Bphy_7808 |  |  |  |  |  |  |
| Burkholderia sp. CCGE1002 | BC1002_6929 | BC1002_6930 | BC1002_6888 | BC1002_6931 | BC1002_6889 | BC1002_6916 |  |
|  |  |  |  |  |  |  |  |
| Burkholderia vietnamiensis G4 | Bcep1808_6015 | Bcep1808_6016 | Bcep1808_6020 | Bcep1808_6017 | Bcep1808_6021 | Bcep1808_5994 |  |
| Burkholderia xenovorans LB400 | Bxe_B1465 | Bxe_B1466 | Bxe_B1470 | Bxe_B1471 | Bxe_B1467 | Bxe_B1446 |  |
| Chlorobaculum parvum NCIB 8327 | Cpar_1619 | Cpar_1622 | Cpar_1624 | Cpar_1623 | Cpar_1625 | Cpar_1626 |  |
| Chlorobium limicola DSM 245 | Clim_0685 | Clim_0682 | Clim_0680 | Clim_0679 | Clim_0681 | Clim_0678 |  |
| Chlorobium phaeobacteroides BS1 | Cphamn1_1751 | Cphamn1_1754 | Cphamn1_1756 | Cphamn1_1755 | Cphamn1_1757 - | Cphamn1_1758 |  |
| Chlorobium tepidum TLS | CT1533 | CT1536 | CT1538 | CT1537 | CT1539 | CT1540 |  |
| Clostridium acetobutylicum ATCC 824 | CAC0253 | CAC0256 | CAC0258 | CAC0257 | CAC0259 | CA_C0259 |  |
| Clostridium beijerinckii NCIMB 8052 | Cbei_0623 | Cbei_2002 | Cbei_2004 | Cbei_2003 | Cbei_2005 | Cbei_2005 |  |
| Clostridium kluyveri DSM 555 | CKL_3081 | CKL_3078 | CKL_3076 | CKL_3077 | CKL_3075 | CKL_3075 | CKL_1746 |
|  | CKL_1748 | CKL_1747 | CKL_1744 | CKL_1745 | CKL_1743 |  | CKL_0371 |
|  |  | CKL_0370 | CLK_0093 | CKL_0372 |  |  |  |
| Cupriavidus taiwanensis | RALTA_p0390 | pRALTA_0391 | pRALTA_0372 | pRALTA_0392 | pRALTA_0373 | pRALTA_0384 |  |
| cyanobacterium UCYN-A | UCYN_06140 | UCYN_06150 | UCYN_06170 | UCYN_06160 | UCYN_06180 | UCYN_06110 |  |
| Cyanothece sp. ATCC 51142 | cce_0559 | cce_0560 | cce_0563 | cce_0561 | cce_0564 | cce_0554 |  |
| Dehalococcoides ethenogenes 195 | DET1158 | DET1155 | DET1153 | DET1154 | DET1152 | DET1148 |  |
| Desulfitobacterium hafniense DCB-2 | Dhaf_0015 | Dhaf_1050 | Dhaf_1052 | Dhaf_1051 | Dhaf_1053 | Dhaf_1056 |  |
| Desulfovibrio vulgaris vulgaris DP4 | Dvul_3090 | Dvul_3093 | Dvul_3096 | Dvul_3094 | Dvul_3097 | Dvul_3098 |  |
| Desulfotomaculum ruminis DSM 2154 | Desru_3454 | Desru_3451 | Desru_3449 | Desru_3450 | Desru_3448 | Desru_3447 |  |
|  |  |  |  |  |  |  |  |
| Frankia alni ACN14a | FRAAL6813 | FRAAL6812 | FRAAL6810 | FRAAL6811 | FRAAL6809 | FRAAL6803 |  |
| Frankia sp. CcI3 | Francci3_4488 | Francci3_4487 | Francci3_4485 | Francci3_4486 | Francci3_4484 | Francci3_4478 |  |
| Geobacter metallireducens GS-15 | Gmet_0662 | Gmet_0663 | Gmet_0669 | Gmet_0664 | Gmet_0669 | Gmet_0681 |  |
| Geobacter sulfurreducens PCA | GSU2821 | GSU2820 | GSU2806 | GSU2819 | GSU2806 | GSU2799 |  |
| Geobacter uraniumreducens Rf4 | Gura_1175 | Gura_1176 | Gura_1201 | Gura_1177 | Gura_1201 | Gura_1209 |  |
| Gluconacetobacter diazotrophicus PAl 5 | GDI0436 | GDI_0437 | GDI_0439 | GDI0438 | GDI0440 | GDI_0430 |  |
| Halorhodospira halophila SL1 | Hhal_0274 | Hhal_0273 | Hhal_0263 | Hhal_0272 | Hhal_0264 | Hhal_0281 |  |
| Heliobacterium modesticaldum Ice1 | HM1_0866 | HM1_0865 | HM1_0863 | HM1_0864 | HM1_0862 | HY04AAS1_0944 |  |
| Herbaspirillum seropedicae SmR1 | Hsero_2853 | Hsero_2852 | Hsero_2850 | Hsero_2851 | Hsero_2849 | Hsero_2870 |  |
|  |  |  |  |  |  |  |  |
| Klebsiella pneumoniae 342 | KPK_1714 | KPK_1713 | KPK_1709 | KPK_1712 | KPK_1708 | KPK_1697 |  |
| Klebsiella variicola At-22 | Kvar_1603 | Kvar_1602 | Kvar_1598 | Kvar_1601 | Kvar_1597 | Kvar_1586 |  |
| Magnetospirillum magneticum AMB-1 | amb1574 | amb1573 | amb1569 | amb1572 | amb1568 | amb1583 |  |
| Mesorhizobium ciceri biovar biserrulae WSM1271 | Mesci_5817 | Mesci_5816 | Mesci_5814 | Mesci_5815 | Mesci_5813 | Mesci_5840 |  |
| Mesorhizobium loti MAFF303099 | mlr5905 | mlr5906 | mlr5908 | mlr5907 | mlr5909 | mll5855 |  |
| Mesorhizobium opportunistum WSM2075 | Mesop_6393 | Mesop_6392 | Mesop_6390 | Mesop_6391 | Mesop_6389 | Mesop_6414 |  |
| Methanobacterium sp. AL-21 | Metbo_1819 | Metbo_2415 | Metbo_2413 | Metbo_2412 | Metbo_2414 | Metbo_0690 | Metbo_1817 |
|  | Metbo_2093 | Metbo_1818 |  | Metbo_1816 |  |  |  |
|  | Metbo_1180 | Metbo_1182 |  |  |  |  |  |
|  |  | Metbo_1181 |  |  |  |  |  |
| Methanothermobacter thermautotrophicus Delta H | MTH1560 | MTH1563 | MTH1565 | MTH1564 | MTH1566 | MTH1871 |  |
| Methanococcus aeolicus Nankai-3 | Maeo_1434 | Maeo_1431 | Maeo_1427 | Maeo_1430 | Maeo_1426 | Maeo_0586 |  |
| Methanococcus maripaludis S2 | MMP0853 | MMP0856 | MMP0858 | MMP0859 | MMP0857 | MMP0658 |  |
|  | MMP0147 |  |  |  |  | MMP0860 |  |
|  |  |  |  |  |  |  |  |
| Methanosarcina acetivorans C2A | MA1205 | MA3898 | MA3900 | MA3899 | MA3901 | MA4195 | MA1209 |
|  | MA1213 | MA1216 |  | MA1218 | MA1219 |  | MA1217 |
|  | MA3895 | MA1210 |  | MA1208 |  |  |  |
| Methanosarcina barkeri fusaro | Mbar_A0171 | Mbar_A0168 | Mbar_A0166 | Mbar_A0167 | Mbar_A0165 | Mbar_A0133 | Mbar_A1552 |
|  |  | Mbar_A2278 | Mbar_A2274 | Mbar_A2276 | Mbar_A2273 |  | Mbar_A2277 |
|  | Mbar_A2281 | Mbar_A1553 |  | Mbar_A1551 |  |  |  |
| Methanosarcina mazei Go1 | MM0719 | MM_0722 | MM_0724 | MM0723 | MM0725 | MM_0757 |  |
|  | Mhun_0280 fused to N |  |  |  |  |  |  |
| Methanothermobacter thermautotrophicus Delta H | MTH1560 | MTH1563 | MTH1565 | MTH1564 | MTH1566 | MTH1871 |  |
|  |  |  |  |  |  |  |  |
| Methylobacterium nodulans ORS 2060 | Mnod_3996 | Mnod_3995 | Mnod_3993 | Mnod_3994 | Mnod_3992 | Mnod_4005 |  |
| Methylobacterium sp. 4-46 | M446_3534 | M446_3535 | M446_3537 | M446_3536 | M446_3538 | M446_3602 |  |
| Methylocella silvestris BL2 | Msil_3632 | Msil_3631 | Msil_3629 | Msil_3630 | Msil_3628 | Msil_3646 |  |
| Methylococcus capsulatus Bath | MCA0229 | MCA0230 | MCA0233 | MCA0231 | MCA0234 | MCA0204 |  |
| Methylomonas methanica MC09 | Metme_1460 | Metme_1461 | Metme_3080 | Metme_1462 | Metme_3081 | Metme_1621 |  |
| 'Nostoc azollae' 0708 | Aazo_1354 | Aazo_1353 | Aazo_1350 | Aazo_1352 | Aazo_1349 | Aazo_1358 |  |
|  | Aazo_3855 |  | Aazo_3531 |  |  |  |  |
|  | Aazo_3771 |  |  |  |  |  |  |
| Nostoc punctiforme PCC 73102 | Npun_F5093 | Npun_R0388 | Npun_F5904 | Npun_R0390 | Npun_R0387 | Npun_R0420 |  |
|  |  |  |  | Npun_F5905 |  |  |  |
| Nostoc sp. PCC 7120 | all1455 | all1454 | all1438 | all1440 | all1437 | all1517 |  |
|  | alr0874 |  | alr3441 |  |  |  |  |
| Pantoea sp. At-9b | Pat9b_4720 | Pat9b_4719 | Pat9b_4715 | Pat9b_4718 | Pat9b_4714 | Pat9b_4703 |  |
| Pelobacter propionicus DSM 2379 | Ppro_3467 | Ppro_3468 | Ppro_3491 | Ppro_3469 | Ppro_3491 | Ppro_3463? |  |
| Polaromonas naphthalenivorans CJ2 | Pnap_2345 | Pnap_2344 | Pnap_2325 | Pnap_2343 | Pnap_2324 | Pnap_1574 |  |
| Prosthecochloris aestuarii DSM 271 | Paes_1627 | Paes_1630 | Paes_1632 | Paes_1631 | Paes_1633 | Paes_1634 |  |
| Pseudomonas stutzeri A1501 | PST_1326 | PST_1327 | PST_1333 | PST_1328 | PST_1334 | PST_1306 |  |
| Rhizobium etli CFN 42 | RHE_PD00202 | RHE_PD00201 | RHE_PD00199 | RHE_PD00200 | RHE_PD00198 | RHE_PD00229 |  |
|  | RHE_PD00308 | RHE_PD00307 |  | RHE_PD00306 |  |  |  |
|  | RHE_PD00260 |  |  |  |  |  |  |
| Rhizobium leguminosarum bv. trifolii WSM1325 | Rleg_4930 | Rleg_4931 | Rleg_4933 | Rleg_4932 | Rleg_4934 | Rleg_4924 |  |
| Rhizobium leguminosarum bv. viciae 3841 | pRL100162 | pRL100161 | pRL100159 | pRL100160 | pRL100158 | pRL100195 |  |
| Rhizobium sp. NGR234 (ANU265) | NGR234_413 | NGR234_414 | NGR234_416 | NGR234_415 | NGR234_417 | NGR234_398 |  |
|  | NGR234_438 | NGR234_439 |  | NGR234_440 |  |  |  |
| Rhodobacter capsulatus SB1003 | RCAP_rcc00572 | RCAP_rcc00571 | RCAP_rcc03280 | RCAP_rcc00570 | RCAP_rcc03279 | RCAP_rcc00566 | RCAP_rcc00587 |
|  | RCAP_rcc00585 | RCAP_rcc00586 |  | RCAP_rcc00588 |  | RCAP_rcc03266 |  |
| Rhodobacter sphaeroides ATCC 17029 | Rsph17029_2192 | Rsph17029_2191 | Rsph17029_2189 | Rsph17029_2190 | Rsph17029_2189 | Rsph17029_2197 |  |
| Rhodomicrobium vannielii ATCC 17100 | Rvan_3363 | Rvan_4619 | Rvan_0507 | Rvan_1955 | Rvan_0506 | Rvan_3444 | Rvan_3361 |
|  | Rvan_1953 | Rvan_3362 | Rvan_1956 | Rvan_0516 | Rvan_1957 | Rvan_3588 | Rvan_0515 |
|  | Rvan_3589 |  | Rvan_0514 | Rvan_3360 |  | Rvan_3361 |  |
|  | Rvan_3556 |  | Rvan_3590 | Rvan_3591 |  | Rvan_0515 |  |
|  |  |  |  | Rvan_0997 |  |  |  |
| Rhodopseudomonas palustris CGA009 | RPA4620 | RPA4619 | RPA4617 | RPA4618 | RPA4616 | RPA4630 | RPA1379 |
|  | RPA1376 | RPA1378 | RPA1373 | RPA1380 | RPA1372 |  | RPA1436 |
|  | RPA1438 | RPA1437 |  | RPA1435 |  |  |  |
| Rhodospirillum centenum SW | RC1_3683 | RC1_3682 | RC1_3680 | RC1_3681 | RC1_3680 | RC1_3690 |  |
| Rhodospirillum rubrum ATCC 11170 | Rru_A1010 | Rru_A1011 | Rru_A2286 | Rru_A1012 | Rru_A2285 | Rru_A0994 | Rru_A1393 |
|  | Rru_A1395 | Rru_A1394 |  | Rru_A1392 |  | Rru_A0796 |  |
| Sinorhizobium fredii NGR234 | NGR_a01130 | NGR_a01120 | NGR_a01100 | NGR_a01110 | NGR_a01090 | NGR_a01270 |  |
|  | NGR_a00890 | NGR_a00880 |  | NGR_a00870 |  |  |  |
| Sinorhizobium medicae WSM419 | Smed_6225 | Smed_6224 | Smed_6222 | Smed_6223 | Smed_6181 | Smed_6233 |  |
| Sinorhizobium meliloti 1021 | SMa0825 | SMa0827 | SMa0830 | SMa0829 | SMa0873 | SMa0814 |  |
| Synechococcus sp. JA-3-3Ab | CYA_1824 | CYA_1823 | CYA_1834 | CYA_1821 | CYA_1835 | CYA_1828 |  |
| Teredinibacter turnerae T7901 | TERTU_1537 | TERTU_1538 | TERTU_1577 | TERTU_1539 | TERTU_1578 | TERTU_1520 |  |
| Trichodesmium erythraeum IMS101 | Tery_4136 | Tery_4137 | Tery_4139 | Tery_4138 | Tery_4139 | Tery_4133 |  |
| Xanthobacter autotrophicus Py2 | Xaut_0088 | Xaut_0089 | Xaut_0091 | Xaut_0090 | Xaut_0092 | Xaut_0111 |  |

**Table S3**

| **Potential diazotrophs** | **Nitrogen fixation genes (locus tag)** | | | | | | |
| --- | --- | --- | --- | --- | --- | --- | --- |
|  | **NifH** | **NifD** | **NifE** | **NifK** | **NifN** | **NifB** | **Anf/VnfG** |
|  |  |  |  |  |  |  |  |
| Alkaliphilus metalliredigens QYMF | Amet_3524 | Amet_3521 | Amet_3519 | Amet_3520 | Amet_3518 | Amet_3517 |  |
| Anaeromyxobacter sp. Fw109-5 | Anae109_3030 | Anae109_3029 | Anae109_3027 | Anae109_3028 | Anae109_3027 | Anae109_3024 |  |
| Anaeromyxobacter sp. K | AnaeK_3203 | AnaeK_3204 | AnaeK_3206 | AnaeK_3205 | AnaeK_3206 | AnaeK_3202 |  |
| Calditerrivibrio nitroreducens DSM 19672 | Calni_0684 | Calni_0685 | Calni_0678 | Calni_0686 | Calni_0679 | Calni_0673 |  |
| Candidatus Accumulibacter phosphatis clade IIA str. UW-1 | CAP2UW1_4447 | CAP2UW1_4448 | CAP2UW1_4373 | CAP2UW1_4372 | CAP2UW1_4449 | CAP2UW1_4422 |  |
| Candidatus Azobacteroides pseudotrichonymphae genomovar. CFP2 | CFPG_545 | CFPG_548 | CFPG_550 | CFPG_549 | CFPG_551 | CFPG_552 |  |
| Candidatus Methanoregula boonei 6A8 | Mboo_1142 | Mboo_1145 | Mboo_1147 | Mboo_1146 | Mboo_1148 | Mboo_0665 |  |
| Candidatus Methanosphaerula palustris E1-9c | Mpal_0561 | Mpal_0558 | Mpal_0556 | Mpal_0557 | Mpal_0555 | Msp_1024 |  |
| Chlorobium chlorochromatii CaD3 | Cag_1244 | Cag_1247 | Cag_1249 | Cag_1248 | Cag_1250 | Cag_1251 |  |
| Chlorobium phaeovibrioides DSM 265 | Cvib_1343 | Cvib_1346 | Cvib_1348 | Cvib_1347 | Cvib_1349 | Cvib_1350 |  |
| Chloroherpeton thalassium ATCC 35110 | Ctha_1035 | Ctha_1032 | Ctha_1030 | Ctha_1031 | Ctha_1029 | Ctha_1028 | Ctha_1832 |
|  | Ctha_1828 | Ctha_1831 |  | Ctha_1833 |  |  |  |
| Clostridium cellulovorans 743B | Clocel_2836 | Clocel_2833 | Clocel_2831 | Clocel_2832 | Clocel_2830 fused to B | Clocel_2830 fused to N |  |
|  | Clocel_3497 |  | Clocel_3496 | Clocel_3495 |  |  |  |
| Clostridium lentocellum DSM 5427 | Clole_0452 | Clole_0455 | Clole_0457 | Clole_0456 | Clole_0458 w/ NifB | Clole_0458 with NifE |  |
|  | Clole_0367 |  |  |  |  |  |  |
| Clostridium ljungdahlii DSM 13528 | CLJU_c23530 | CLJU_c04930 | CLJU_c04950 | CLJU_c04940 | CLJU_c04960 | CLJU_c04960 |  |
|  | CLJU_c04900 |  | CLJU_c23520 | CLJU_c23510 |  | CLJU_c23540 |  |
|  | CLJU_c23120 |  | CLJU_c23030 |  |  |  |  |
|  | CLJU_c23050 |  | CLJU_c23110 |  |  |  |  |
| Coraliomargarita akajimensis DSM 45221 | Caka_2839 | Caka_2838 | Caka_2836 | Caka_2837 | Caka_2835 | Caka_2834 |  |
| Dechloromonas aromatica RCB | Daro_1415 | Daro_1414 | Daro_1501 | Daro_1413 | Daro_1505 | Daro_1454 |  |
| Denitrovibrio acetiphilus N2460, DSM 12809 | Dacet_1046 | Dacet_1047 | Dacet_1050 | Dacet_1048 | Dacet_1051 | Dacet_1055 |  |
| Desulfarculus baarsii Konstanz, DSM 2075 | Deba_0443 | Deba_0440 | Deba_0436 | Deba_0439 | Deba_0435 | Deba_0434 |  |
|  |  |  |  |  |  | Deba_0438 |  |
| Desulfatibacillum alkenivorans AK-01 | Dalk_1522 | Dalk_1519 | Dalk_1517 | Dalk_1518 | Dalk_1516 | Dalk_1515 |  |
| Desulfobacca acetoxidans DSM 11109 | Desac_0350 | Desac_0353 | Desac_0356 | Desac_0354 | Desac_0357 | Desac_0358 |  |
|  |  |  |  |  |  | Desac_0578 |  |
| Desulfobacterium autotrophicum HRM2 | HRM2_09670 | HRM2_09700 | HRM2_09720 | HRM2_09710 | HRM2_09730 | HRM2_09740 |  |
| Desulfomicrobium baculatum DSM 4028 | Dbac_0834 | Dbac_0837 | Dbac_0841 | Dbac_0838 | Dbac_0842 | Dbac_0843 |  |
| Desulfotomaculum acetoxidans DSM 771 | Dtox_1023 | Dtox_1026 | Dtox_1028 | Dtox_1027 | Dtox_1029 | Dtox_1030 |  |
| Desulfotomaculum reducens MI-1 | Dred_2821 | Dred_2818 | Dred_2816 | Dred_2817 | Dred_2815 | Dred_2814 |  |
|  |  |  |  |  |  |  |  |
| Desulfovibrio magneticus RS-1 | DMR_20560 | DMR_20530 | DMR_17520 | DMR_20520 | DMR_17510 | DMR_17500 |  |
| Desulfovibrio salexigens DSM 2638 | Desal_0447 | Desal_0444 | Desal_0439 | Desal_0443 | Desal_0438 | Desal_0437 |  |
|  |  |  |  |  |  | Desal_0442 |  |
| Desulfobulbus propionicus DSM 2032 | Despr_3052 | Despr_3055 | Despr_2706 | Despr_3056 | Despr_2705 | Despr_2704 |  |
| Desulfotomaculum carboxydivorans CO-1-SRB | Desca_1134 | Desca_1137 | Desca_1139 | Desca_1138 | Desca_1140 | Desca_1141 |  |
| Desulfotomaculum kuznetsovii DSM 6115 | Desku_3346 | Desku_3343 | Desku_3341 | Desku_3342 |  | Desku_3340 |  |
|  | Desku_2205 | Desku_2204 |  |  |  |  |  |
|  |  | Desku_2207 |  |  |  |  |  |
| Desulfurispirillum indicum S5 | Selin_0690 | Selin_0691 | Selin_0262 | Selin_0692 | Selin_0263 | Selin_2076 |  |
| Desulfurivibrio alkaliphilus AHT2 | DaAHT2_2455 | DaAHT2_2458 | DaAHT2_2472 | DaAHT2_2459 | DaAHT2_2476 | DaAHT2_2477 |  |
| Desulfovibrio aespoeensis Aspo-2 | Daes_1021 | Daes_1024 | Daes_1028 | Daes_1025 | Daes_1029 | Daes_1030 |  |
|  |  |  |  |  |  | Daes_1026 |  |
| Desulfurivibrio alkaliphilus AHT2 | DVUA0015 | DaAHT2_2458 | DaAHT2_2472 | DaAHT2_2459 | DaAHT2_2476 | DaAHT2_2477 |  |
| Dickeya dadantii Ech703 | Dd703_0491 | Dd703_0492 | Dd703_0497 | Dd703_0493 | Dd703_0498 | Dd703_0513 | Dd703_2612 |
|  | Dd703_2610 | Dd703_2611 |  | Dd703_2613 |  |  |  |
| Ethanoligenens harbinense YUAN-3 | Ethha_1567 | Ethha_1564 | Ethha_1562 | Ethha_1563 | Ethha_1561 w/ NifB | Ethha_1561- w/NifE | Ethha_2313 |
|  | Ethha_2309 | Ethha_2312 |  | Ethha_2314 |  |  |  |
|  |  | Ethha_1721 |  |  |  |  |  |
|  |  | Ethha_2317 |  |  |  |  |  |
|  |  | Ethha_2316 |  |  |  |  |  |
| Erwinia carotovora atroseptica SCRI1043 | ECA2956 | ECA2955 | ECA2949 | ECA2954 | ECA2948 | ECA2937 |  |
| Geobacter bemidjiensis Bem | Gbem_2074 | Gbem_2075 | Gbem_2077 | Gbem_2076 | Gbem_2077 | Gbem_2083 |  |
| Geobacter lovleyi SZ | Glov_0650 | Glov_0649 | Glov_0639 | Glov_0648 | Glov_0639 | Glov_0422 |  |
| Geobacter sp. FRC-32 | Geob_2458 | Geob_2575 | Geob_2581 | Geob_2577 | Geob_2581 | Geob_2456 |  |
|  |  |  |  |  |  | Geob_2591 |  |
| Hydrogenobacter thermophilus TK-6 | Hydth_1128 | Hydth_1129 | Hydth_1134 | Hydth_1130 | Hydth_1135 | Hydth_1114? |  |
| Hyphomicrobium sp. MC1 | HYPMC_3687 | HYPMC_3686 | HYPMC_3684 | HYPMC_3685 | HYPMC_3683 | HYPMC_3666 |  |
|  | HYPMC_3637 |  |  |  |  |  |  |
| Ilyobacter polytropus CuHBu1, DSM 2926 | Ilyop_1410 | Ilyop_1407 | Ilyop_1405 | Ilyop_1406 | Ilyop_1404 | Ilyop_1402 |  |
| Leptothrix cholodnii SP-6 | Lcho_1430 | Lcho_1431 | Lcho_1345 | Lcho_1432 | Lcho_1350 | Lcho_1404 |  |
| Magnetococcus sp. MC-1 | Mmc1_1202 | Mmc1_1201 | Mmc1_1195 | Mmc1_1200 | Mmc1_1194 | Mmc1_1206 |  |
| Methanocella sp. RC-I | RCIX1606 | RCIX1611 | RCIX1614 | RCIX1613 | RCIX1616 | RC1X325 |  |
| Methanococcus vannielii SB | Mevan_0065 | Mevan_0062 | Mevan_0060 | Mevan_0059 | Mevan_0061 | Mevan_1533 |  |
| Methanoplanus petrolearius SEBR 4847, DSM 11571 | Mpet_0263 | Mpet_0260 | Mpet_0258 | Mpet_0259 | Mpet_0257 | Mpet_1949? |  |
| Methanosaeta concilii GP6 | MCON_0607 | MCON_0610 | MCON_0612 | MCON_0611 | MCON_0613 | MCON_0910 |  |
|  | MCON_0503 |  |  |  |  |  |  |
|  | Mhun_0280 fused to N |  |  |  |  |  |  |
| Methanothermobacter marburgensis str. Marburg | MTBMA_c01460 | MTBMA_c01490 | MTBMA_c01510 | MTBMA_c01500 | MTBMA_c01520 | MTBMA_c04420 |  |
|  | MTBMA_c10230 |  | MTBMA_c00640 | MTBMA_c01510 |  |  |  |
|  |  |  | MTBMA_c01050 |  |  |  |  |
| Methanothermococcus okinawensis IH1 | Metok_1120 | Metok_1117 | Metok_1114 | Metok_1116 | Metok_1113 | Metok_1108 |  |
|  | Metok_0807 |  |  |  |  |  |  |
| Methylacidiphilum infernorum V4 | Minf_1876 | Minf_1874 | Minf_1872 | Minf_1873 | Minf_1871 | Minf_0453 |  |
| Paludibacter propionicigenes WB4 | Palpr_1366 | Palpr_1426 | Palpr_1430 | Palpr_1427 | Palpr_1431 | Palpr_1432 | Palpr_1362 |
|  | Palpr_1423 | Palpr_1363 |  | Palpr_1361 |  | Palpr_1362 |  |
| Pectobacterium atrosepticum SCRI1043 | ECA2956 | ECA2955 | ECA2949 | ECA2954 | ECA2948 | ECA2937 |  |
| Pelobacter carbinolicus DSM 2380 | Pcar_2098 | Pcar_2099 | Pcar_0313 | Pcar_2100 | Pcar_0314 | [Pcar_2107](http://www.ncbi.nlm.nih.gov/entrez/query.fcgi?db=gene&cmd=Retrieve&dopt=full_report&list_uids=3724798) |  |
| Pelodictyon luteolum DSM 273 | Plut_1528 | Plut_1531 | Plut_1533 | Plut_1532 | Plut_1534 | Plut_1535 |  |
| Pelodictyon phaeoclathratiforme BU-1 | Ppha_1949 | Ppha_1952 | Ppha_1954 | Ppha_1953 | Ppha_1955 | Ppha_1956 |  |
| Sideroxydans lithotrophicus ES-1 | Slit_0881 | Slit_0882 | Slit_0903 | Slit_0883 | Slit_0904 | Slit_0837 |  |
| Spirochaeta smaragdinae SEBR 4228, DSM 11293 | Spirs_3008 | Spirs_3005 | Spirs_3003 | Spirs_3004 | Spirs_3002 | Spirs_3001 |  |
| Spirochaeta thermophila DSM 6192 | STHERM_c03530 | STHERM_c03500 | STHERM_c03480 | STHERM_c03490 | STHERM_c03470 | STHERM_c03460 |  |
| Sulfuricurvum kujiense DSM 16994 | Sulku_1500 | Sulku_1498 | Sulku_1289 | Sulku_1497 | Sulku_1297 | Sulku_1510 |  |
|  |  |  |  |  |  |  |  |
| Syntrophobacter fumaroxidans MPOB | Sfum_1019 | Sfum_1016 | Sfum_1013 | Sfum_1015 | Sfum_1012 | Sfum_1011 |  |
| Syntrophobotulus glycolicus DSM 8271 | Sgly_2853 | Sgly_2840 | Sgly_2836 | Sgly_2839 | Sgly_2835 | Sgly_2834 | Sgly_2849 |
|  | Sgly_2841 | Sgly_2850 |  | Sgly_2848 |  | Sgly_2810 |  |
| Thermincola potens JR | TherJR_0688 | TherJR_0691 | TherJR_0693 | TherJR_0692 | TherJR_0692 | TherJR_0695 |  |
| Thermoanaerobacterium thermosaccharolyticum DSM 571 | Tthe_1866 | Tthe_1865 | Tthe_1860 | Tthe_1864 | Tthe_1859 | Tthe_1867 |  |
| Thermocrinis albus HI 11/12, DSM 14484 | Thal_0424 | Thal_0425 | Thal_0430 | Thal_0426 | Thal_0431 | Thal_0413 |  |
| Thermodesulfovibrio yellowstonii DSM 11347 | THEYE_A1717 | THEYE_A1716 | THEYE_A1714 | THEYE_A1715 | THEYE_A1714 | THEYE_A1710 |  |
| Tolumonas auensis DSM 9187 | Tola_0650 | Tola_0651 | Tola_0655 | Tola_0652 | Tola_0656 | Tola_0604 |  |
| Wolinella succinogenes DSM 1740 | WS1394 | WS1392 | WS1390 | WS1388 | WS1391 | WS1397 |  |
|  |  |  |  |  |  |  |  |
| Zymomonas mobilis mobilis ZM4 | ZMO1823 | ZMO1824 | ZMO1826 | ZMO1825 | ZMO1827 | Za10_1418 |  |

**Table S4**

| **Species containing C-type enzymes** | **Nitrogen fixation genes (locus tag)** | | | | | |
| --- | --- | --- | --- | --- | --- | --- |
|  | **NifH** | **NifD** | **NifE** | **NifK** | **NifN** | **NifB** |
| Caldicellulosiruptor hydrothermalis 108 | Calhy_2145 | Calhy_2140 | Calhy_2138 | Calhy_2139 |  | Calhy_2137 |
| Caldicellulosiruptor kristjanssonii 177R1B | Calkr_1798 | Calkr_1795 | Calkr_1793 | Calkr_1794 |  | Calkr_1792 |
| Caldicellulosiruptor kronotskyensis 2002 | Calkro_2123 | Calkro_2118 | Calkro_2120 | Calkro_2119 |  | Calkro_2117 |
| Caldicellulosiruptor saccharolyticus | Csac_2466 | Csac_2463 | Csac_2461 | Csac_2462 |  | Csac_2460 |
| Candidatus Desulforudis audaxviator MP104C | Daud_0143 | Daud_0146 | Daud_0148 | Daud_0147 |  | Daud_0149 |
| Methanocaldococcus infernus ME | Metin_0041 | Metin_0038 | Metin_0033 | Metin_0037 |  | Metin_0554 |
|  | Metin_0054 |  |  |  |  |  |
| Methanocaldococcus sp. FS406-22 | MFS40622_0031 | MFS40622_0034 | MFS40622_0041 | MFS40622_0035 |  | MFS40622_0164 |
|  | MFS40622_1279 |  |  |  |  |  |
| Methanocaldococcus vulcanius M7 | Metvu_1081 | Metvu_1084 | Metvu_0866 | Metvu_1085 |  | Metvu_0123 |
|  | Metvu_0884 |  |  |  |  |  |
| Roseiflexus castenholzii DSM 13941 | Rcas_4043 | Rcas_4041 |  | Rcas_4040 |  | Rcas_4042 |
| Roseiflexus sp. RS-1 | RoseRS_1201 | RoseRS_1199 |  | RoseRS_1198 |  | RoseRS_1200 |
| Syntrophothermus lipocalidus DSM 12680 | Slip_2130 | Slip_2127 | Slip_2125 | Slip_2126 |  | Slip_2124? |
| Thermodesulfatator indicus DSM 15286 | Thein_1049 | Thein_1053 |  | Thein_1054 |  | Thein_1052 |
|  |  | Thein_1056 |  |  |  |  |
