# Supplementary material for: Distribution of nitrogen fixation and nitrogenase-like sequences amongst microbial genomes
Source: BMC Genomics. 2012 May 3;13:162. doi: 10.1186/1471-2164-13-162 (PMC3464626; doi:10.1186/1471-2164-13-162)
Supplement: Additional file 3 — Figure S1. Neighbor joining phylogenetic tree of the Nif/Vnf/AnfD and K sequences derived from the species shown in Figure 3. [file 1471-2164-13-162-S3.doc]

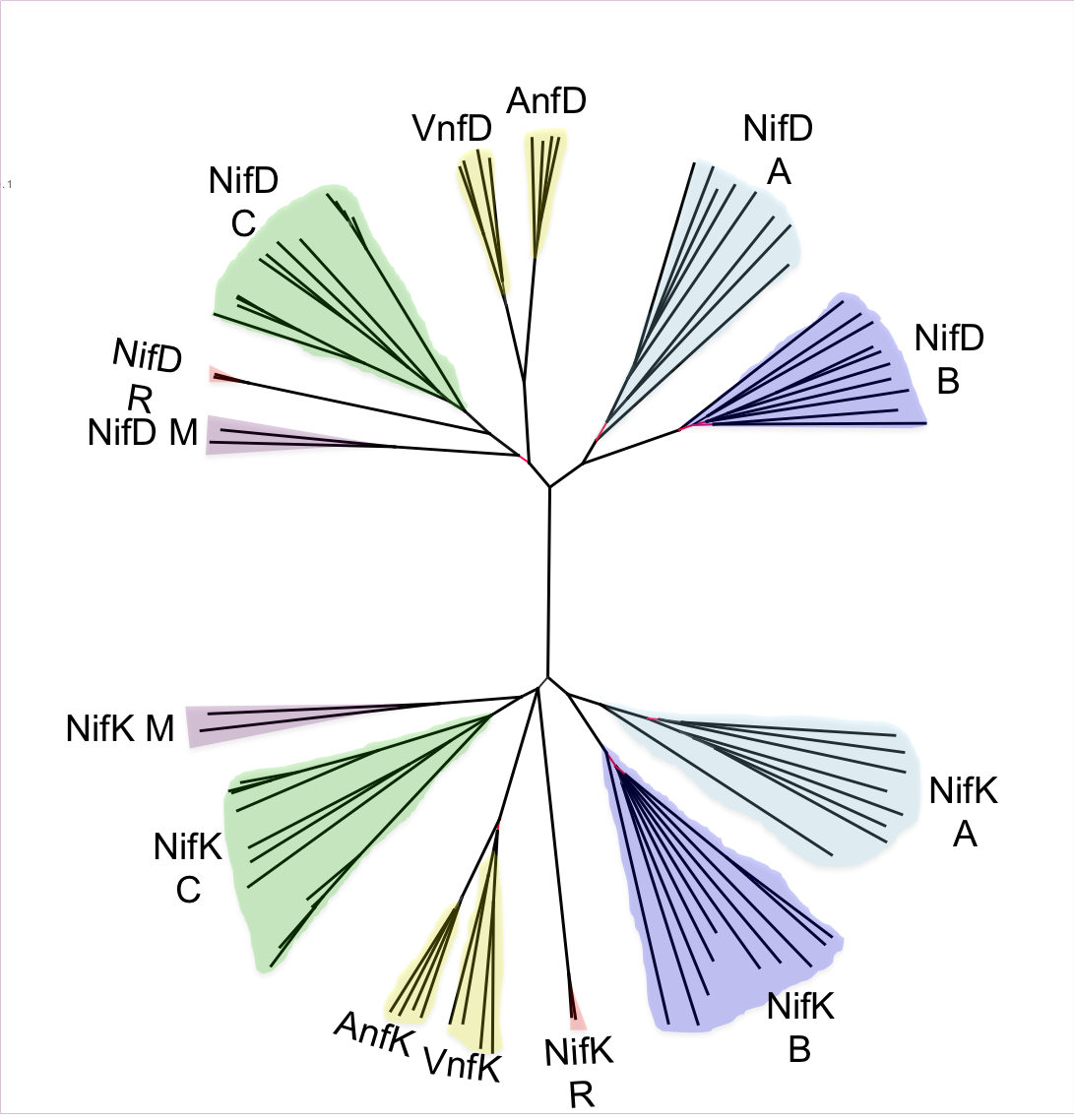


Figure S1. Neighbor joining phylogenetic tree of the Nif/Vnf/AnfD and K sequences derived from the species shown in Figure 3. Clades are highlighted to indicate the number and diversity of sequences in each clade. The sub-families A, B and C of NifD and NifK, derived from the alignments of NifD sequences in Fig 3 with additional alignments of NifK sequences are indicated. The phylogenetic analysis divides group C into two further clades: M, comprising *Methanobacterium thermoautotrophicum* Delta H, and *Methanococcus maripaludis* S2 and R, comprising *Roseiflexus castenholzii* and *Roseiflexus* sp. Branches colored light red represent low bootstrap confidence (50-60%). Phylogeny was evaluated with Splitstree version 4.11.3 [36] by applying the BioNJ algorithm on ProteinML distances computed using the WAG evolutionary model. Bootstrap proportions were obtained from 1,000 replicates.
