# Supplementary material for: Distribution of nitrogen fixation and nitrogenase-like sequences amongst microbial genomes
Source: BMC Genomics. 2012 May 3;13:162. doi: 10.1186/1471-2164-13-162 (PMC3464626; doi:10.1186/1471-2164-13-162)
Supplement: Additional file 4 — Figure S2. Gene neighborhoods of selected nitrogenase-like proteins. [file 1471-2164-13-162-S4.doc]

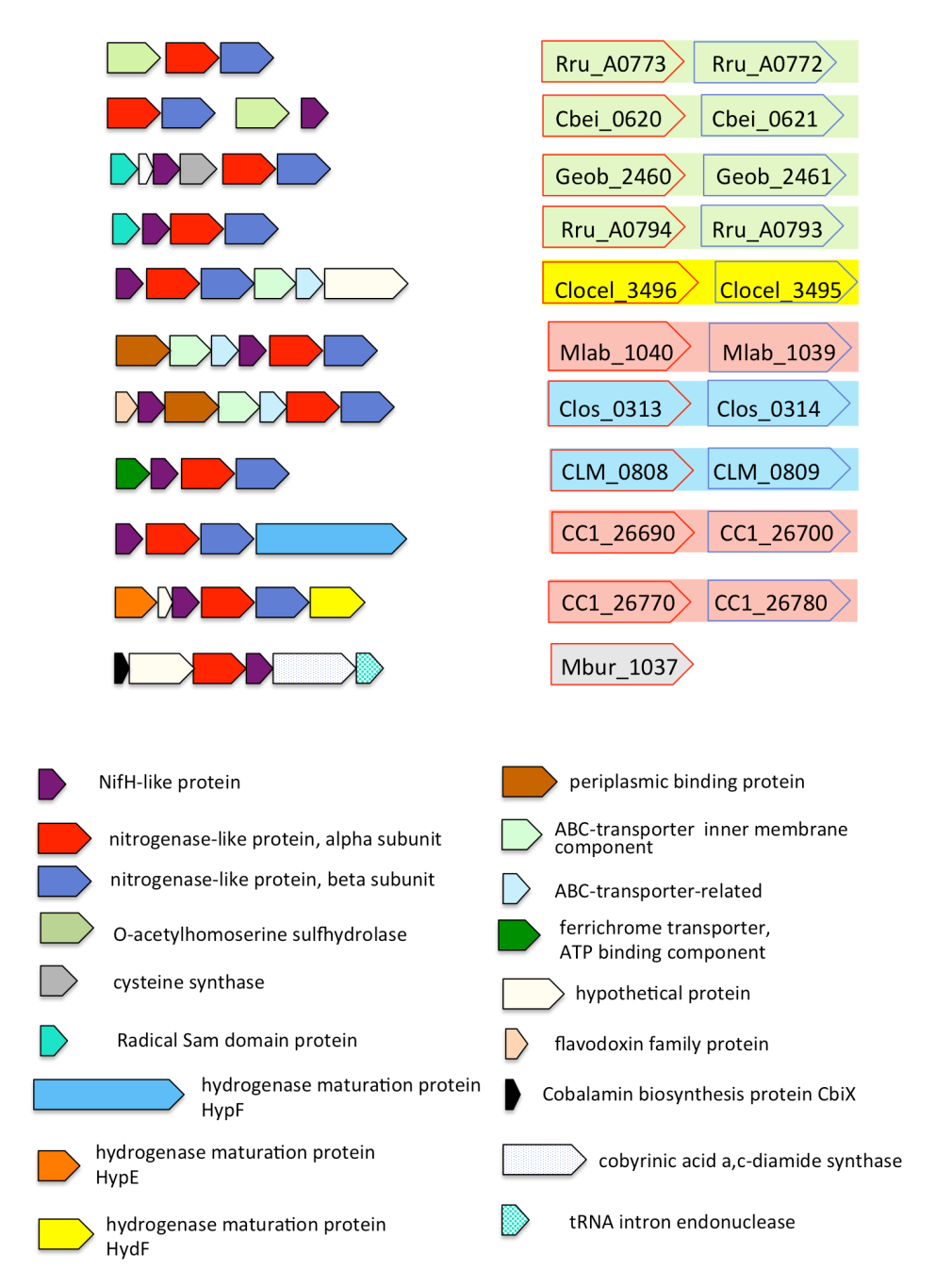


Figure S2. Gene neighborhoods of selected nitrogenase-like proteins. Neighborhoods are depicted at the top left-hand side of the figure, color-coded according to the function key shown below. Locus-tags for the corresponding alpha subunit (red) and beta subunit (dark blue) of each nitrogenase-like protein are indicated on the right of each neighborhood. Locus tags are color-coded according to the phylogenetic groupings shown in Fig 4.
